# Wnt5A signaling supports antigen processing and CD8 T cell activation

**DOI:** 10.3389/fimmu.2022.960060

**Published:** 2022-08-26

**Authors:** Tresa Rani Sarraf, Malini Sen

**Affiliations:** Cancer Biology and Inflammatory Disorder, Council of Scientific & Industrial Research -Indian Institute of Chemical Biology, Kolkata, India

**Keywords:** actin, antigen, CD8, proteasome, wnt5A

## Abstract

Antigen processing and antigen-specific CD8 T cell activation form part and parcel of cell-mediated immunity to infections. Yet, several lacunae remain in our understanding of how antigen processing and CD8 T cell response are coordinated. In this study, using mouse bone marrow-derived dendritic cells (BMDC) as antigen-presenting cells and Ovalbumin (OVA)/DQ-Ovalbumin (DQ-OVA) as model antigen we demonstrated that Wnt5A signaling in BMDC supports antigen processing/presentation and concomitant CD8 T cell activation through regulation of actin and proteasome dynamics. Recombinant Wnt5A conditioning of BMDC and associated actin assembly facilitated DQ-OVA processing, which was inhibited by the proteasome inhibitor MG132. Moreover, Wnt5A depletion led to a significant reduction in OVA processing and presentation. Impaired DQ-OVA processing in Wnt5A depleted BMDC correlated with altered dynamics of both actin and the proteasome regulator PA28α-PA28β, and reduced association of DQ-OVA with actin and proteasome subunits. Inhibited OVA processing/presentation in the Wnt5A depleted BMDC also resulted in subdued activation of OVA-sensitized CD8 T cells in co-culture with the BMDC. In concurrence with these findings, we demonstrated reduced OVA processing and impaired CD8 T cell response to OVA immunization in Wnt5A heterozygous mice lacking a copy of the Wnt5A gene in comparison to the wild-type cohorts. Taken together, our results reveal a crucial requirement of Wnt5A signaling in antigen processing/presentation and CD8 T cell activation, thus unveiling a vital regulatory node of cell-mediated immunity, unidentified thus far.

## Introduction

Antigen processing, its cross-presentation to CD8 T cells, and antigen-specific CD8 T cell expansion constitute a vital feature of cell-mediated immunity to viral and other microbial infections. Quite naturally, impairments in any of these processes lead to increased susceptibility to infections. Yet, several gaps remain in our understanding of how host intracellular processes engage toward the activation and preservation of antigen-specific CD8 T cell responses (1–3). We previously demonstrated that Wnt5A signaling supports pathogen killing through phagocytosis and xenophagy, by regulating actin assembly (4–6). Considering that Wnt5A signaling is associated with cell and organelle polarity (7, 8), it is not surprising that Wnt5A signaling is a major regulator of the cytoskeletal actin network, which constitutes the fundamental framework for organelle crosstalk (4–6, 9–11). Since antigen processing is associated with actin remodeling (12), it was important to investigate if Wnt5A signaling also promotes antigen processing and presentation, thereby controlling T cell activation and cell-mediated immunity.

Wnt5A belongs to a large family of secreted Wnt glycoprotein ligands that signal upon binding to Frizzled and/or ROR cell surface receptors (13–16). Wnt-Frizzled/ROR signal transduction occurs in various cell types ranging from epithelial and mesenchymal cells to specialized cells of the immune system including lymphocytes, macrophages, and dendritic cells (14, 17–19). On account of sequence homology among the different Wnt ligands and their receptors, these signaling entities function in multiple combinations under different physiological conditions. The mode of Wnt-Frizzled/ROR signaling in a particular cell type in fact depends to a great extent on the existing ligand-receptor stoichiometry and the availability of the signaling intermediates (20). While the canonical mode of Wnt signaling is commonly associated with the transcriptional coactivator β-catenin, the non-canonical mode, of which Wnt5A signaling is an example, is able to drive intracellular processes independent of β-catenin; although crosstalk with canonical signaling intermediates is not uncommon (21).

In the current study, using ovalbumin (OVA) as a model foreign antigen and mouse bone marrow-derived dendritic cells (BMDC) as antigen-presenting cells we demonstrated how a Wnt5A-Actin axis promotes antigen processing and concomitant activation of antigen-responsive CD8 T cells. Furthermore, we depicted that the occurrence of partial Wnt5A gene silencing in Wnt5A heterozygous (Wnt5A+/-) mice causes considerably subdued OVA-specific CD8 T cell recall response following immunization with OVA, indicating that Wnt5A signaling is crucial for antigen processing/presentation, CD8 T cell activation and sustenance of antigen-specific CD8 T cell memory.

## Materials and methods

The list of reagents is summarized in **Supplementary Table 1**.

### Animal maintenance

Breeding and maintenance of B6;129S7-*Wnt5a^tm1Amc^*/J mice (Wnt5A+/+ and Wnt5A+/-), purchased from Jackson Laboratory, USA, and BALB/c mice were carried out in the institutional animal facility. Characterization of Wnt5A wild-type and Wnt5A heterozygous mice for experimental purposes were accomplished by genotyping, following Jackson laboratory protocol [https://www.jax.org/Protocol?stockNumber=004758&protocolID=23556]. All animals were maintained in optimum physiological conditions with balanced light and dark cycles in IVC (Individually Ventilated Caging) system with *ad libitum* water and food. All experimental and control mice were 8-10 weeks old. Both male and female mice were used in all animal experiments described here and similar male: female ratios were maintained in experimental and control sets.

### Ethics approval

Animal Ethics Committee of CSIR-IICB (CSIR-IICB-AEC) approved all animal studies in its meeting held on 19^th^ September 2019.

### Mice immunization

Mice were immunized following published protocols with some modifications (22–24). In brief, OVA (100ug) dissolved in 100ul PBS, and an equal volume of CFA or IFA were mixed to form an emulsion and each mouse was injected subcutaneously with the emulsion at the tail base at day 0 (OVA + CFA) and day 14 (OVA + IFA). For the immunization of the B6;129S7-*Wnt5a^tm1Amc^*/J mice 20ug SIINFEKL peptide was used with 80ug OVA. Mice were sacrificed for assay 8 – 10 days after the second dose of immunization.

### BMDC generation

BMDC were derived from the bone marrow of the femur and tibia of BALB/c or C57BL/6 mice following published protocols (25, 26). In brief, cells collected from the bone marrow were suspended in RPMI supplemented with 10% FBS, 2 mM glutamine, 1 μg ml^-1^ streptomycin, 1-unit ml^-1^ penicillin, 20 ng/ml mouse recombinant GM-CSF and 10 ng/ml mouse recombinant IL-4, following RBC lysis and washing, and cultured in bacterial plates under normal tissue culture ambience. On the 3^rd^ day, 50% of the culture medium was replaced by fresh medium and on the 6^th^ day, cells were harvested for experiments.

### BMDC transfection

Wnt5A siRNA and control/scramble siRNA were used for transfecting BMDC using the reverse transfection method (27). In brief, for transfection in each well of a 6-well tissue culture plate, siRNA (25nM) and lipofectamine RNAimax were diluted separately in 150μl OptiMEM each and mixed gently. The 300ul mix was incubated for 15 min. at room temperature, following which harvested BMDC in OptiMEM (1X10^5^ cells/300ul) were mixed separately with either Wnt5A siRNA-RNAimax or control siRNA-RNAimax in a total volume of 1ml. 1ml cells were plated in each well of a 6-well tissue culture plate. The transfected cell medium was replaced by RPMI supplemented with 10% FBS, 24 hr. post-transfection, and incubation was continued for 24 hr.-26 hr. until assay.

### RNA isolation and cDNA preparation

RNA isolation from cells was done using the Trizol reagent. cDNA was prepared by the cDNA synthesis kit from BioBharati Life Sciences Kolkata, following instructions provided by the manufacturer. PCR was done using the following pairs of primers: Wnt5A (forward 5’- CAGGTCAACAGCCGCTTCAAC-3’ and reverse 5’- ACAATCTCCGTGCACTT CTTGC-3’), GAPDH (forward 5’-ACCACAGTCCATGCCATCAC-3’ and reverse 5’-TCCACCACCCTGTTGCTGTA-3’), MHCI (forward 5’-CGCACAGAGATACTACAACC-3’ and reverse 5’-ATCTGGGAGAGACAGATCAG -3’).

### Western blotting

BMDC were harvested for lysis 48 hr. post-transfection. Cell suspension in lysis buffer (150mM NaCl, 50mM Tris-Cl pH 8.0, 0.1% SDS, 1% Triton X-100, 0.5% sodium deoxycholate, 50mM DTT, 1mM EDTA, 5% glycerol, 5mM NaF, 2mM Na_3_VO_4_, 50mM PMSF and Protease Inhibitor Cocktail) was incubated on ice for 20 min. following the centrifugation at 12,000 rpm for 10 min. at 4°C and SDS-PAGE was run with about 10ug of protein. After PVDF membrane transfer, blocking was performed with 5%BSA in TBST followed by primary antibody incubation at 4°C overnight. After washing with TBST 3X, the blot was incubated with HRP conjugated secondary antibody for 2 hr. at room temperature. Chemiluminescence reagent was used for developing the blot in chemidoc. Band intensities were measured by GelQuant software.

### ELISA

The level of secreted Wnt5A by BMDC was measured by coating ELISA plate wells with 100ul of BMDC conditioned media. After washing with PBST (0.05% Tween in PBS) wells were blocked with 1% BSA for 2 hr. at room temperature, and washed thereafter. Subsequently, after incubation with Wnt5A primary antibody overnight at 4°C, wells were washed again with PBST and incubated with HRP conjugated secondary antibody for 2 hr. at room temperature. TMB substrate was used for developing the reaction. The reaction was stopped immediately after color development (15 – 30 minutes) by 250mM HCL and absorbance was measured at 450nm in an ELISA reader.

### Confocal microscopy

#### Estimation of DQ-OVA and phalloidin intensity

For estimation of DQ-OVA intensity, washed and dried poly-l-lysine coated chamber slides were used for culturing BMDC under different conditions. Following treatment with rWnt5A (100ng/ml) or PBS (vehicle control) for 6 hr., or 47 hr. post-transfection with Wnt5A siRNA/control siRNA, BMDC were pulsed with DQ-OVA (5ug/ml) for 45 min., counterstained with DAPI, washed thereafter with cold PBS 3X, fixed in 3% paraformaldehyde for 15 min. at room temperature and washed again with PBS 3X. Additionally, rWnt5A treated or transfected BMDC were stained with phalloidin (diluted 1:2000 in PBST with 2.5% BSA) for 15 min., counterstained with DAPI (1:3000) for 10 min., and washed with PBST 3X before evaluation of phalloidin intensity. Transfected BMDC were separately incubated with CD11c antibody at room temperature for 1 hr., counterstained with DAPI, and washed with PBS 3X to evaluate the extent of purity by confocal microscopy. For analysis of the effect of MG132 on DQ-OVA processing, rWnt5A pretreated BMDC were incubated with MG132 (10uM) 15 min. after DQ-OVA pulsing, and incubation was continued for 30 min. before harvesting. In separate experiments, BMDC treated with rWnt5A (100ng/ml) for 6 hr. either with or without RAC1 inhibitor (15uM) or Disheveled inhibitor (15uM) for the last 3 hr. were washed and stained with phalloidin. All treated samples were mounted in 60% glycerol or prolong glass antifade for confocal microscopy using Leica TCS-SP8 confocal microscope [63X magnification (1X or 2X zoom)].

#### Estimation of PA28α/β and 20sα intensity

For performing PA28α/β staining or 20sα staining, transfection and DQ-OVA pulsing of BMDC were carried out on chamber slides. Subsequently, after fixation in 3% paraformaldehyde, cells were permeabilized for 10 min. at room temperature by 0.1% Triton X -1%BSA in PBS, washed with PBS 3X, and blocked for 1 hr. at room temperature with 1% BSA in PBST. Treated BMDC were incubated overnight at 4°C separately with either PA28α, PA28β, or 20sα primary antibody diluted in PBST with 1% BSA. Next day cells were washed with PBS 3X and incubated with secondary antibody (diluted in 1% BSA) for 2 hr. at room temperature. After counterstaining with DAPI for 10 min. at room temperature, the cells were washed 3X with PBS and mounted in prolong glass antifade for confocal microscopy using Leica TCS-SP8 confocal microscope [63X magnification (2X zoom)]. ImageJ (NIH) software was used to measure the fluorescence intensity of each cell.

#### BMDC and T cell adherence

For estimating T cells attached to BMDC transfected with either Wnt5A siRNA or control, the chamber slide grown BMDC were incubated with ovalbumin (100ug/ml) about 28 hr. after transfection, and incubation was continued for another 20 hr. After washing, the OVA pulsed transfected BMDC were co-cultured with MACs purified CD8 T cells from ovalbumin immunized mice for 1 hr. at 37°C. After washing with PBS 3X to remove unbound T cells, samples were fixed in 3% paraformaldehyde for 15 min. at room temperature. BMDC-T cell conjugate was analyzed by confocal microscopy. In some experiments, anti-CD11c and anti-CD8 staining were performed for identifying BMDC and CD8 T cells. Samples were mounted in 60% glycerol for visualization of BMDC-T cell conjugates by confocal microscopy [63X magnification (3X or 2X zoom) of Leica TCS-SP8 confocal microscope].

### Flow cytometry

#### OVA uptake and processing

For estimation of antigen uptake and antigen processing, transfected BMDC (47 hr. post-transfection) were pulsed with either FITC-Ova (5ug/ml) or DQ-OVA (5ug/ml) for 45 min., after which cells were washed with cold PBS and stained for 1 hr. with anti-CD11c antibody for subsequent FACS (BD.LSR Fortessa Cell analyzer). In separate experiments, BMDC pretreated with rWnt5A (100ng/ml) for 6 hr. were pulsed with DQ-OVA for 45 min., and Arp2 inhibitor (20uM) or DMSO (vehicle control) was added for the last 30 min. incubation. Harvested cells were stained with anti-CD11c antibody and processed for FACS analysis.

For comparing DQ-OVA processing in Wnt5A+/+ and Wnt5A+/- mice, a CD11c enriched population of cells was separated from harvested splenocytes by MACs column following instructions provided by the manufacturer, to focus on CD11c expressing dendritic cells/macrophages and other phagocytes. The column-separated CD11c enriched cells were pulsed with DQ-OVA for 45 min., washed, and stained for 1hr. with anti-CD11c antibody. Subsequently, 7AAD was added for the detection of dead cells before FACS acquisition.

#### Phalloidin staining

Wnt5A siRNA and control siRNA transfected BMDC were harvested 48 hr. post-transfection, washed with 1XPBS, and stained with anti-CD11c antibody for 1hr. at 4°C. Cells were further washed and stained with phalloidin (diluted 1:100 in PBST with 2.5% BSA) for 15 min. After additional washing cells were analyzed by FACS.

#### MHC Class I/Class II and SIINFEKL-MHC Class I conjugate expression

Transfected BMDC (BALB/c mice generated), either stimulated with OVA (100ug/ml) for 20 hr. or left un-stimulated, were harvested, washed with PBS with 0.5% BSA, and stained with anti-CD11c, anti-MHCI (H-2K[d]), and anti-MHCII (I-A/I-E) for 1 hr. at 4°C. After washing cells were analyzed by FACS. In separate experiments similarly treated BMDC (C57BL/6 mice generated), both stimulated with OVA and un-stimulated were stained with anti-CD11c and anti-Mouse H-2Kb-SIINFEKL (25D1.16) antibody before processing for FACS analysis.

#### CD8 T cell proliferation

For analysis of the proliferation of CD8 T cells in coculture with BMDC through CFSE dye dilution, CD8 T cells were purified from total spleen and lymph node of OVA immunized or unimmunized mice by MACs column mediated negative selection after RBC lysis (cshprotocols.cshlp.org/content/2006/1/pdb.rec390), and thereafter labeled with 2uM CFSE in PBS for 10 min. at 37°C (https://flowcytometry.utoronto.ca/wp-content/uploads/2016/01/Proliferation-CFSE.pdf), before co-culturing with BMDC (either pulsed or not pulsed with 100ug/ml OVA). CD8 T cell purity and CFSE labeling were confirmed by FACS, before setting up the co-culture. After 4 days of co-culture, floating and loosely adherent cells were harvested, stained with anti-CD3 and anti-CD8 antibodies for 1 hr. at 4°C, for validation of CD8 T cells and PI or 7AAD was added before FACS acquisition for excluding dead cells. The proliferation of CD8 T cells upon exposure to OVA pulsed BMDC was estimated from the extent of CFSE dilution, using CFSE dilution in the “no OVA” set as a reference. For measuring OVA recall response in Wnt5A+/+ and Wnt5A+/- mice by CFSE dye dilution, cells harvested from mice spleens and lymph nodes were labeled with CFSE after RBC lysis. Following incubation with OVA (100ug/ml) for 4 days, cells were harvested and stained with anti-CD3 and anti-CD8 antibody for 1 hr. at 4°C. In a subset of similar experiments, MHCI tetramer staining was performed before CD8 and CD3 staining. For all samples either PI or 7AAD was used as cell viability dye before FACS acquisition. Proliferation was estimated in the same way as described earlier through CFSE dye dilution.

#### Intracellular cytokine expression

Intracellular cytokines were measured after 48 hr. of culture following Brefeldin (5ug/ml) treatment for the last 4 hr. Harvested cells were fixed with 1% paraformaldehyde for 15 min. at room temperature, permeabilized with 0.1% Tween-20-1%BSA-PBS for 10 min. at room temperature, washed with PBST 3X, and finally stained with anti-CD3, anti-CD8, anti-IL2, anti-IFNγ, and anti-Granzyme B antibodies in PBST (PBS + 0.05%Tween) with 1% BSA, for 1 hr. at 4°C, before FACS acquisition.

#### Statistical analysis

Statistical analysis was performed using Student’s t-test. Student’s paired t-test was performed for densitometry units, MFI of DQ-OVA, MFI of MHCI, MFI of MHCII, % of proliferated CD8 T cells, ELISA, and adherent T cells per 100 DC for Wnt5A siRNA (Wsi) and control siRNA (Csi) transfected BMDC. The student’s unpaired t-test was performed for intensity and signal overlap measurement by confocal microscopy and FACS analysis involving Wnt5A wild-type and heterozygous mice.

## Results

### Wnt5A promotes the processing of exogenous antigen in association with actin assembly

Given the association of Wnt5A signaling with cytoskeletal actin dynamics, a player in intracellular organelle trafficking and phagocytosis (4–6, 8, 28) we investigated if a Wnt5A-Actin axis influences antigen processing, this being a prerequisite for antigen presentation and T cell activation. To this end, we used mouse bone marrow-derived dendritic cells (BMDC) as model antigen processing/presenting cells and ovalbumin (OVA)/DQ-OVA as a model antigen.

The bone marrow of BALB/c mice was used to generate BMDC following published protocols (25, 26), and the purity was confirmed by the flow cytometry using the CD11c antibody (**Figure S1A**). We demonstrated that BMDC expresses both the secreted ligand Wnt5A as well as Frizzled5, an established receptor for Wnt5A (28–30), indicating that BMDC is capable of both paracrine and autocrine Wnt5A signaling (**Figures S1B, C**). In order to evaluate the influence of Wnt5A signaling on OVA processing, we treated BMDC culture with BODIPY conjugated DQ-Ovalbumin (DQ-OVA), which emits bright fluorescence while being proteolytically processed. The extent of antigen processing, in the form of cleaved DQ-OVA, was evaluated by the intensity of the green and red fluorescence emitted depending on the different stages of processing (31–33). Treatment of BMDC with DQ-OVA for 45 min. resulted in optimal fluorescence (**Figure S2A**).

**Figure 1:**
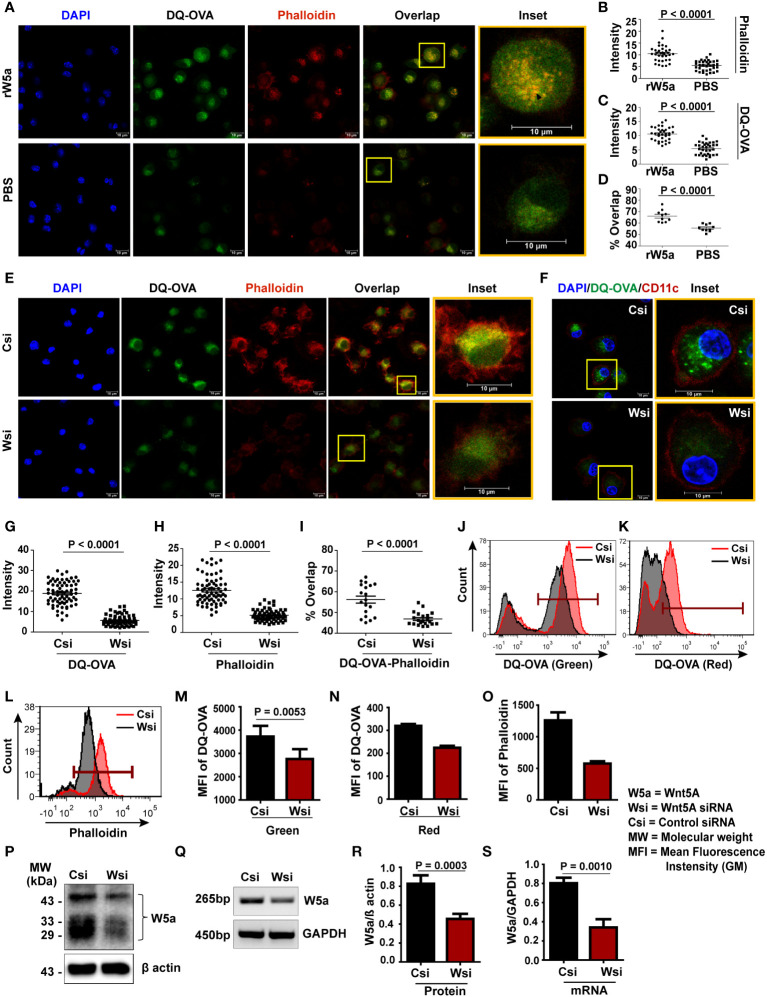
Wnt5A promotes DQ-OVA processing in BMDC: **(A–D)** rWnt5A mediated increase in phalloidin-red intensity **(A, B)**, DQ-OVA-green intensity **(A**, **C)**, and DQ-OVA-phalloidin overlap intensity **(A**, **D)** in comparison to vehicle control (PBS), as demonstrated by confocal microscopy of conditioned (rWnt5A treated) BMDC, and subsequent ImageJ analysis (intensity measurement) and Mander’s overlap coefficient analysis of different microscopic fields. Merge/overlap of DQ-OVA and phalloidin is emphasized in insets. The nucleus is denoted by the DAPI stain. **(E, G–I)**: Similar representations demonstrating correlative decreases in DQ-OVA intensity, phalloidin intensity, and DQ-OVA-phalloidin overlap intensity in Wsi (siRNA transfected BMDC) compared to Csi (control siRNA transfected BMDC). The BMDC marker CD11c expression in OVA processing BMDC is demonstrated in **F, J-O**: Representation of flow cytometry **(J-L)** and MFI (Geometric Mean Fluorescence Intensity) plot **(M-O)** on CD11c gated cells demonstrating a decrease in DQ-OVA and phalloidin fluorescence in Wnt5A depleted BMDC (Wsi) as compared to control (Csi). About 50% depletion of Wnt5A by transfection was detected at both protein and mRNA levels (**P, Q** respectively, cropped from raw data, as explained in S3) and by corresponding densitometry **(R, S)**. For intensity measurement related to confocal microscopy **(B, C, G, H)**, each dot represents a cell from two independent experiments (n = 2, ~14 fields). For Mander’s overlap coefficient analysis (**D, I**: n = 2, 10 fields), each dot represents a field from two independent experiments. Magnification: 63X (2X zoom). The arrow mark (black) shows overlap (yellow spots). For flow cytometry (n = 2 - 5) marker gate for DQ-OVA was selected based on an unstained cell histogram. The bar represents mean ± SEM (Standard error of the mean) with exact p values depicted in the figure. n represents the number of individual experiments. For **(B–D, G–I)** Student’s unpaired t-test was performed and for **J** – **O** and **R**, **S** Student’s paired t-test was performed.

Initially, we demonstrated an increased association of assembled actin with increased DQ-OVA processing in recombinant Wnt5A (rWnt5A) treated BMDC as compared to the corresponding PBS control. **Figure 1A** is a confocal microscopic representation of higher intensities of phalloidin-red (a measure of assembled filamentous actin), DQ-OVA-green, and DQ-OVA-phalloidin (overlap)-yellow in rWnt5A treated BMDC than the vehicle (PBS) treated control. DAPI (blue) denotes the cell nuclei. **Figures 1B-D** are quantitative representations of the rWnt5A-induced increases in phalloidin intensity, DQ-OVA intensity, and DQ-OVA-phalloidin overlap intensity respectively. This observation indicates that Wnt5A signaling coordinates actin assembly to support antigen processing. In essence, Wnt5A supports actin assembly in BMDC through its signaling intermediates Disheveled and RAC1 as clearly depicted by the use of Disheveled and RAC1 inhibitors in **Figures S3A, B**. Moreover, the involvement of actin assembly in antigen processing is corroborated by its blockade through the application of an inhibitor of Arp2/3 activity, which is essential for actin assembly (**Figures S4A, B**) (34). To further validate the dependence of antigen processing on the Wnt5A-Actin axis we examined the effect of siRNA-mediated silencing of Wnt5A on DQ-OVA processing. Accordingly, both Wnt5A siRNA transfected and scramble siRNA (control) transfected BMDC were treated with DQ-OVA for about 45 min. to enable OVA internalization and processing, following which intracellular fluorescence of both phalloidin and DQ-OVA was measured. Indeed, confocal microscopy revealed significantly lower fluorescence intensities of DQ-OVA, phalloidin, and DQ-OVA – phalloidin overlap in the Wnt5A siRNA sets than in the corresponding controls (**Figure 1E**). CD11c expression, specific for BMDC is depicted separately in **Figures 1F**. **Figures 1G**–**I** depict the numeric differences in the respective fluorescence (DQ-OVA/phalloidin) and fluorescence overlap (DQ-OVA – phalloidin) intensities between the Wnt5A depleted (Wnt5A siRNA transfected) cells and the corresponding controls. Flow cytometry separately conducted on DQ-OVA treated BMDC, transfected with either Wnt5A siRNA or control siRNA yielded results that were very similar to those yielded by confocal microscopy (**Figures 1J-O**). The degree of DQ-OVA processing (shown by two different laser channels, FITC and PE-Texas red) corresponding to different stages of processing (33), was significantly less in the Wnt5A depleted BMDC than in the corresponding control (J, K, M, N). Additionally, decreased DQ-OVA intensity in the Wnt5A depleted BMDC correlated with a substantial decrease in the intensity of phalloidin (L, O), thus corroborating that Wnt5A signaling in association with actin dynamics is required for antigen processing. **Figures 1P-S** and **S5A-B** represent the siRNA-mediated reduction (about 50%) in Wnt5A expression in BMDC, both at the mRNA and protein levels. **Figures S5A-B** demonstrates the raw data pertaining to **Figure 1P–S**. There was no significant influence of Wnt5A on OVA uptake (**Figure S6**), showing that the observed effect of Wnt5A on OVA processing is not due to any notable alteration in OVA uptake.

### Wnt5A-assisted antigen processing/presentation is proteasome-mediated

**Figure 2:**
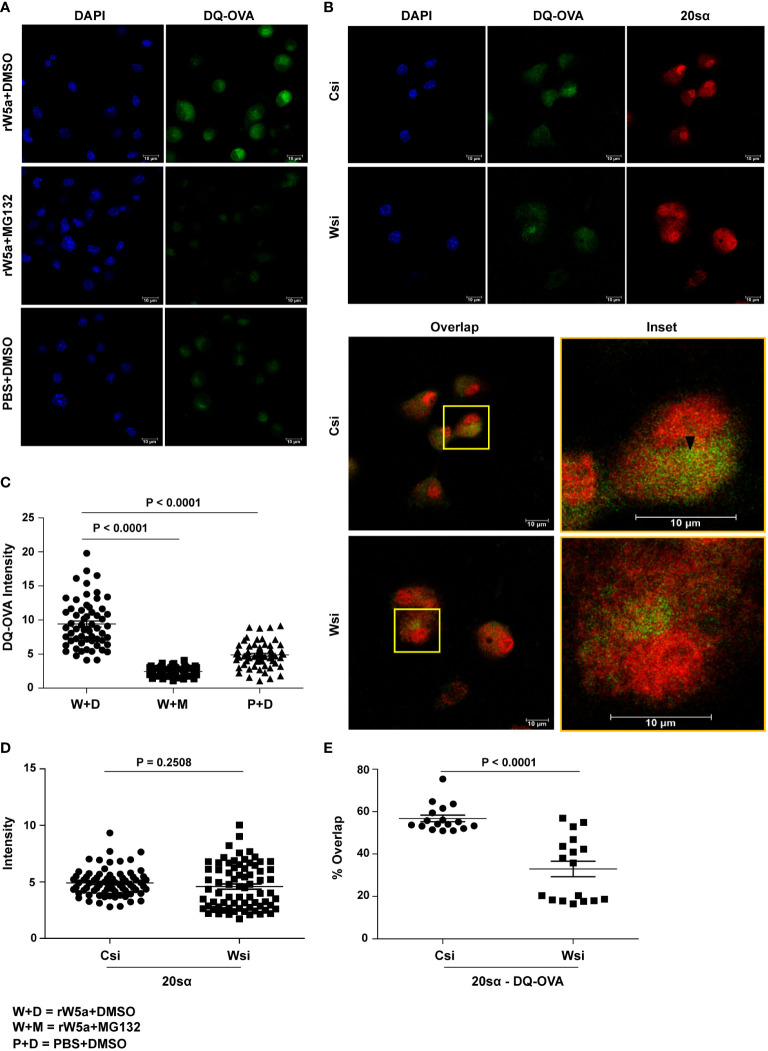
Wnt5A mediated DQ-OVA processing in BMDC is associated with proteasome activity: **(A, C)**: Confocal microscopy of rWnt5A (rW5A) conditioned BMDC samples with or without MG132 **(A)** and corresponding ImageJ analysis of DQ-OVA intensity **(C)** (n = 2, each dot representing a cell) demonstrate MG132 mediated blockade in rW5a induced increase in DQ-OVA fluorescence. PBS (P) and DMSO (D) are vehicle controls for rW5a (W) and MG132 (M) respectively. DAPI (blue) fluorescence denotes the nuclei. B, D, and E: Confocal microscopy **(B)** along with ImageJ intensity measurement **(D)** (n = 2, each dot representing a cell) and Mander’s overlap coefficient analysis **(E)** (n = 2, 17 fields, each dot representing a field) demonstrate that with no significant change in 20S proteasome subunit α intensity in Wsi (Wnt5A depleted) BMDC as compared to Csi (control), there is a significant decrease in the DQ-OVA-20Sα overlap intensity indicating reduced DQ-OVA processing. Overlap of DQ-OVA with the 20Sα is emphasized in inset **(B)**. The arrow mark (black) shows overlap (yellow spots). Magnification: 63X (2X zoom). The bar represents mean ± SEM (Standard error of the mean) with exact p values depicted in the figure. n represents the number of independent experiments. For all calculations, Student’s Unpaired t-test was performed.

The fact that DQ-OVA processing in BMDC is to a large extent inhibited by MG132, a proteasomal inhibitor, and not so much by NH4Cl, a lysosomal inhibitor (35–37) (**Figure S7A, B**), prompted us to investigate if Wnt5A assisted DQ-OVA processing is linked with proteasome activity. Indeed, rWnt5A induced an increase in DQ-OVA processing in BMDC was significantly reduced by the application of MG132 as revealed by the estimation of fluorescence by confocal microscopy (**Figures 2A, C**). Additional validation of proteasomal involvement in Wnt5A assisted antigen (DQ-OVA) processing was made by demonstrating decreased association (overlap: orange/yellow) of DQ-OVA (green) with the 20sα core subunit (red) of the proteasome in Wnt5A knocked down (Wnt5A siRNA: Wsi) BMDC as compared to control (scramble siRNA: Csi) by confocal microscopy and Mander’s overlap coefficient analysis (38, 39). Overlap is emphasized in the inset and marked by an arrowhead. Wnt5A depletion did not cause any significant alteration in 20sα expression as judged by ImageJ intensity measurement (**Figures 2B, D, E**).

**Figure 3:**
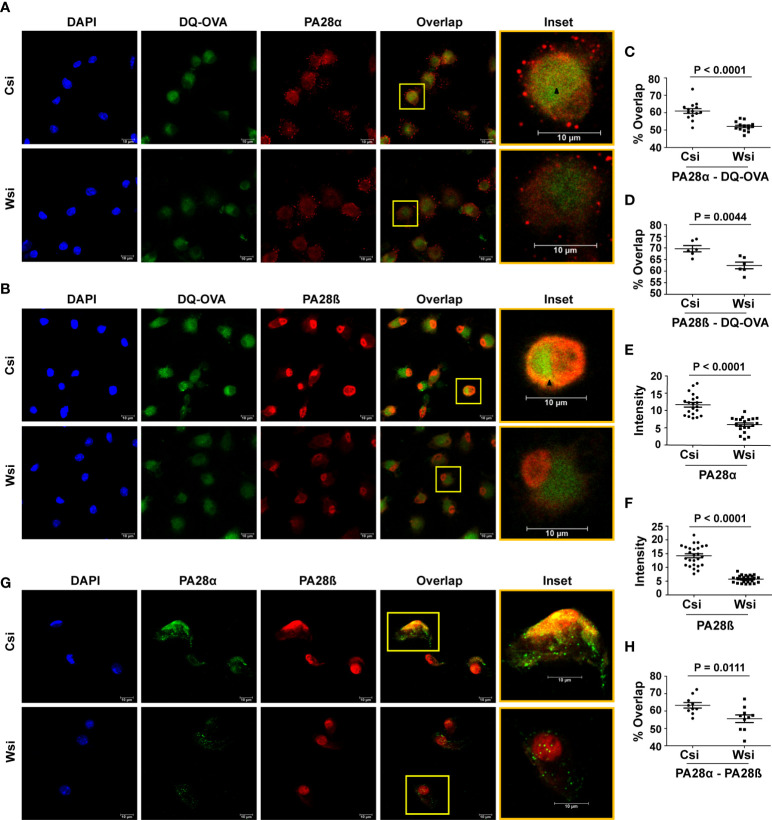
Wnt5A mediated DQ-OVA processing is associated with the proteasome regulator PA28α-PA28β: Confocal microscopy **(A, B)** along with Mander’s overlap coefficient analysis **(C, D)** n = 2, 6-14 fields, each dot representing a field and ImageJ intensity measurement **(E, F)** n = 3, 7-8 fields, each dot representing a cell, demonstrate a significant decrease in DQ-OVA-PA28α and DQ-OVA-PA28β overlap, as well as reduced individual intensities of PA28α and PA28β respectively in Wnt5A depleted (Wsi) BMDC as compared to control (Csi). **(G**, **H)**: Confocal microscopy of Wsi or Csi BMDC **(G)** and Mander’s overlap coefficient analysis **(H)** (n = 2, 10 fields, each dot representing a field) demonstrate reduced PA28α − PA28β overlap in Wsi transfected BMDC as compared to control (Csi). Magnification: 63X (2X zoom). The arrow mark (black) shows overlap (yellow spots). The bar represents mean ± SEM (Standard error of the mean) with exact p values depicted in the figure. n represents the number of independent experiments. For all calculations, Student’s Unpaired t-test was performed.

In view of the involvement of the proteasome regulator PA28α-PA28β hetero-oligomer with the processing/presentation of antigens including OVA (40–42), we further examined if Wnt5A assisted OVA antigen processing is associated with PA28α and PA28β. Initially, we demonstrated proteasomal involvement of DQ-OVA processing by colocalization/overlap of fluorescing DQ-OVA and PA28β in BMDC (**Figure S7C**). Subsequently, we demonstrated significantly reduced overlap (yellow/orange) of both PA28α (red) and PA28β (red) with fluorescing DQ-OVA (green) in BMDC sets where Wnt5A was depleted by Wnt5A siRNA (Wsi), in comparison to the control/scramble siRNA sets (Csi), by confocal microscopy (**Figures 3A-D**). Overlap (yellow/orange) is emphasized in the inset by an arrow mark. Wnt5A depletion in fact also led to a significant reduction in the levels of both PA28α and PA28β (**Figures 3A, B, E, F**). These results indicate that Wnt5A signaling regulates both the expression of the PA28 proteasome regulator subunits as well as their association with the antigen being processed. Upon examining the effect of siRNA-mediated Wnt5A depletion on PA28α-PA28β co-localization by confocal microscopy, furthermore, we found that PA28α (green)-PA28β (red) overlap (yellow/orange) was significantly reduced in Wnt5A depleted BMDC sets as compared to the corresponding controls, implying that Wnt5A signaling also regulates PA28α-PA28β assembly on the proteasome (**Figures 3G, H**). The extent of overlap (PA28α/PA28β/-DQ-OVA and PA28α-PA28β) was measured using Manders overlap coefficient (39). Similar results were obtained using Pearson’s coefficient (43). Taken together, our experimental results reveal that Wnt5A signaling promotes antigen processing by acting in conjunction with actin and proteasome dynamics, which is also regulated independent of antigen by Wnt5A signaling.

In compliance with the MHC Class I molecule-mediated cell surface presentation of proteasome processed antigens (40, 44) and the observed requirement of Wnt5A signaling for proteasome-mediated antigen (OVA) processing, we found reduced MHC Class I surface expression in Wnt5A depleted (Wnt5A siRNA transfected: Wsi) OVA pulsed BMDC as compared to the corresponding control (Csi) (**Figure S8A**). Impaired OVA processing by depletion of Wnt5A signaling could be the cause of reduced surface presentation of stable MHCI-OVA peptide conjugates. There was no detectable change in the level of MHC Class I (α-chain) mRNA expression in BMDC upon depletion of Wnt5A (**Figure S8B**), indicating that the observed reduction in MHC Class I surface expression was not due to a change in gene expression. Wnt5A depletion in BMDC did not affect the surface expression of MHC Class II, which is involved in the presentation of endosomal antigens (**Figure S8C**), in line with the observed ineffectiveness of NH4Cl, a lysosomal/endosomal inhibitor on DQ-OVA processing in BMDC. The requirement of Wnt5A for MHCI mediated surface presentation of OVA antigen was separately validated in OVA pulsed BMDC generated from C57BL/6 instead of BALB/c mice. Initially, Wnt5A-dependent processing of DQ-OVA in the C57BL/6 derived BMDC was demonstrated by the inhibitory effect of siRNA-mediated Wnt5A reduction on DQ-OVA processing (**Figures S9A, B**). Wnt5A depleted (Wnt5A siRNA: Wsi) BMDC pulsed with OVA were subsequently shown to express significantly reduced cell surface MHCI-SIINFEKL conjugates in comparison to the corresponding control as estimated by FACS with anti-H-2Kb-SIINFEKL antibody (**Figures S9C, D**). Accordingly, it was important to examine if Wnt5A facilitated proteasome-mediated antigen processing/presentation is directly linked with MHC Class I restricted CD8 T cell activation. Since notable Wnt5A dependency of MHCII surface expression upon antigenic stimulation was not observed initially, evaluation of MHCII restricted CD4 T cell activation was not included in this study.

### Wnt5A controlled antigen-processing supports CD8 T cell response

In order to examine if Wnt5A-actin-proteasome activity supports MHC Class I restricted antigen-specific CD8 T cell response, we used OVA pulsed BMDC (with or without siRNA mediated Wnt5A reduction) for antigen recognition by OVA-sensitized CD8 T cells harvested from OVA immunized BALB/c mice. Herein, we followed published reports where antigen-sensitized CD8 T cells harvested from immunized mice have been shown to proliferate upon major histocompatibility complex matched exposure to cells pulsed with the same antigen ex vivo (45–48). Following co-culturing with BMDC, CD8 T cell proliferation was estimated by CFSE dilution (49) and CD8 T cell activation was estimated by checking intracellular IL2 and Granzyme B (GRB) levels (50–52).

**Figure 4:**
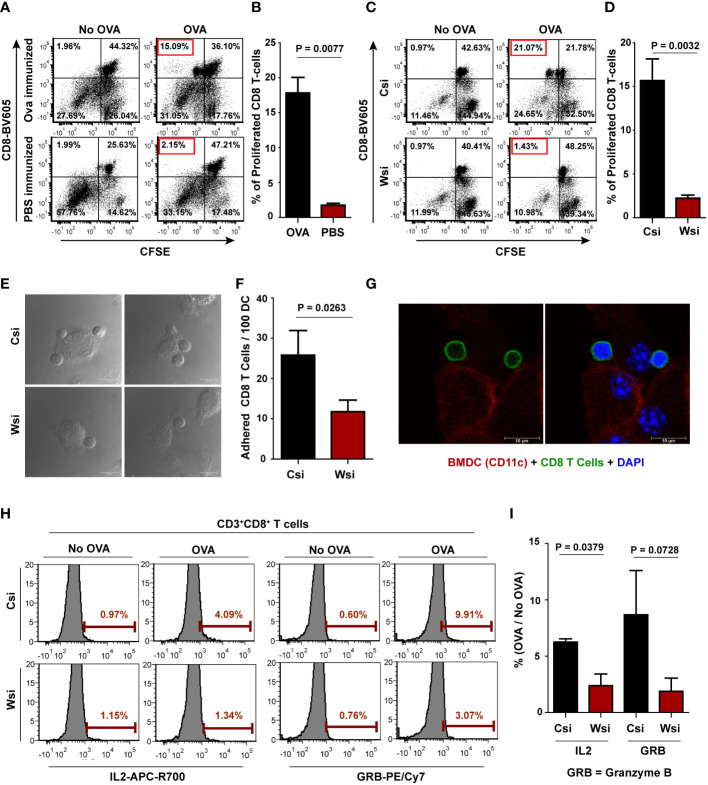
Wnt5A depletion in BMDC causes diminished CD8 T cell response in OVA pulsed BMDC – CD8 T cell coculture: **(A, B)**: Representative FACS dot plot showing significantly less proliferation (as identified by CFSE less population) of CD8 T cells harvested from OVA unimmunized (PBS+CFA/IFA injected) mice as opposed to those harvested from OVA immunized mice **(A)** upon exposure to OVA pulsed BMDC and its graphical analysis **(B)** (n = 4). For each proliferation experiment, BMDC without OVA pulsing was used as a reference. **(C**, **D)**: Representation of proliferation of CD8 T cells harvested from OVA immunized mice upon exposure to OVA pulsed BMDC (Wsi or Csi) **(C)** and its graphical analysis **(D)** (n = 5), revealing significantly less T cell response to Wnt5A depleted (Wsi) BMDC. **(E, F)**: CD8 T cells in coculture adhere less to OVA pulsed Wsi-BMDC as compared to OVA pulsed control (Csi-BMDC) as revealed by confocal microscopy (n = 3). **(G)**: Confocal microscopy demonstrating adhered CD8 T cells (CD8: green) to BMDC (CD11c: red) (n = 2). Magnification: 63X (3X or 2X zoom). **(H, I)**: Representative examples of OVA-specific % positive CD8 T cells (compared to no-OVA) with high intracellular IL2 (APC-R700), and GRB (PE-Cyanine 7) in co-culture with OVA pulsed BMDC (Wsi vs Csi) as analyzed by flow cytometry **(H)** and the overall graphical representation of stimulation index (%OVA/%no-OVA) **(I)** (n = 3), showing Wnt5A facilitated CD8 T cell response in OVA pulsed BMDC-CD8 T cell coculture. Marker gate for highly positive cells of the OVA sets for IL2 and GRB was based on the corresponding no OVA control. An unstained cell histogram of each cytokine was used to determine the population of IL2 and GRB producers. The bar represents mean ± SEM (Standard error of the mean) with exact p values depicted in the figure. n represents the number of individual experiments. For B, D, F, and I Student’s paired t-test was performed.

The efficacy of OVA-sensitized CD8 T cell generation by OVA immunization was initially validated by demonstrating that CD8 T cells harvested from OVA immunized mice and labeled with CFSE proliferate much more efficiently than similarly treated CD8 T cells from OVA unimmunized (only PBS-CFA/IFA injected) mice upon exposure to OVA pulsed BMDC. CFSE labeled CD8 T cells exposed to BMDC without OVA pulsing (no OVA) were used as a reference for these experiments. The extent of proliferation of CD8 T cells (from OVA immunized or unimmunized mice) in response to OVA pulsed BMDC in a 4-day co-culture was measured by estimating the CFSE dye diluted (CFSE^dim^) population delineated based on the extent of CFSE dye dilution in the ‘no OVA’ reference (**Figures 4A, B**). Thereafter, the role of Wnt5A-assisted antigen processing/presentation in CD8 T cell response was evaluated by examining the effect of Wnt5A depletion in OVA pulsed BMDC on OVA-sensitized CD8 T cell proliferation and activation, as described below. The purity of isolated CD8 T cells is depicted in (**Figure S10A**). The CD3+CD8+CFSE^dim^ lymphocytes were gated by excluding dead cells, as depicted in **Figure S10B**.

CD8 T cells harvested from the spleen and lymph nodes of OVA immunized BALB/c mice were labeled with CFSE and placed in co-culture with BALB/c derived OVA pulsed BMDC transfected with either Wnt5A siRNA or scramble siRNA (control) before OVA pulsing. A portion of the BMDC, either transfected with Wnt5A siRNA or scramble siRNA, but not stimulated with OVA was placed in a similar co-culture with CFSE labeled CD8 T cells to serve as “no OVA” un-stimulated reference for each “Wnt5A siRNA-BMDC-OVA” co-culture set or “scramble siRNA-BMDC-OVA” co-culture set. After 4 days of co-culture cells were harvested and CD8 T cell proliferation was estimated after flow cytometry from the extent of dilution of CFSE intensity (49), as represented in **Figures 4C, D**. For both Wnt5A siRNA (Wsi)-BMDC-T cell co-culture and control/scramble (Csi)-BMDC-T cell co-culture, cells were gated based on the expression of CD3 and CD8 after live-dead cell gating as depicted in **Figure S10C**. In compliance with the observed reduction in OVA-specific CD8 T cell response, Wnt5A reduction correlated with a considerably lower number of CD8 T cells adhering to BMDC about 1 hr. after co-culture, implying subdued CD8 T cell recognition by the BMDC plausibly on account of impaired OVA processing and presentation (**Figures 4E, F**). Association between BMDC (CD11c: red) and CD8 T cell (CD8: green) was validated separately by confocal microscopy (**Figure 4G**). CD8 T cell activation was evaluated by comparing the levels of intracellular IL2 and granzyme B. Impaired OVA-specific CD8 T cell activation by Wnt5A knocked down BMDC was reflected by the reduced percentages of IL2^high^ and granzyme B^high^ CD8 T cells in the Wnt5A siRNA-BMDC-CD8 T cell coculture in comparison to control, as depicted in the FACS representation (**Figures 4H, I**) corresponding to a 48 hr. co-culture. BMDC-"no OVA"-CD8 T cell co-culture was used as a reference for each BMDC-OVA-CD8 T cell co-culture. **Figure S10D** depicts the gating conducted for FACS analysis. As a whole, these results indicate that Wnt5A signaling in antigen-presenting cells supports MHCI-restricted CD8 T cell activation and expansion.

### Antigen-specific CD8 T cell activation is impaired in Wnt5A heterozygous (Wnt5A+/-) background

**Figure 5:**
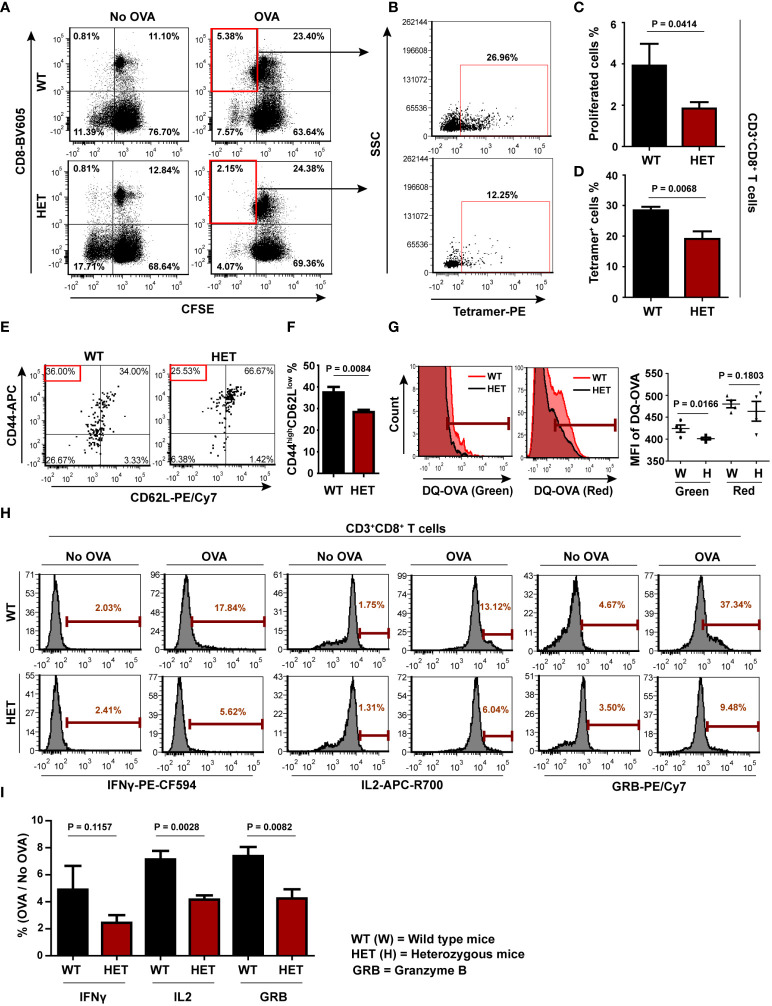
CD8 T cell recall response to OVA is impaired in Wnt5A heterozygous (Wnt5A +/-) mice: **(A)**: Significantly less CD8 T cell proliferative response in splenocyte cultures of OVA immunized Wnt5A heterozygous (HET) mice in response to added OVA in comparison to that of the corresponding wild-type cohorts (WT) is demonstrated by CFSE dye dilution through FACS. Less OVA responsive CD8 T cell proliferation in case of Wnt5A heterozygous mice correlates with a reduced percentage of SIINFEKL-MHCI tetramer binding CD8 T cells **(B)**. **(C**, **D)**: Graphical estimate of the percentage of OVA responsive proliferating CD8 T cells and SIINFEKL-MHCI tetramer binding cells in OVA immunized Wnt5A heterozygous and wild-type mice (for **C**, n=7 and for **D** n=4 n representing the number of mice in each group). Higher percentages of SIINFEKL MHCI-tetramer (PE) binding CD8 T cells in WT than in HET correlate with a relatively higher degree of effector phenotype [CD44high (APC) CD62Llow (PE-CY7)] in the WT **(E, F)** (n = 4). Quadrant setup was based on the secondary control of CD62L and unstained cells. **(G)** Representative FACS histogram and distribution plot show less DQ-OVA processing in splenic CD11c positive cells of Wnt5A heterozygous mice as compared to those of Wnt5A wild-type mice in both FITC and PE-Texas red channels (n = 4). **(H**, **I)**: Representative histograms of % OVA responsive CD8 T cells (compared to no-OVA stimulation) with high intracellular IL2 (APC-R700), IFNγ (PE-CF594) and GRB (PE-Cyanine 7) levels as demonstrated by FACS of splenocytes of OVA immunized WT and HET mice **(H)** and the overall projection of stimulation index (%OVA/%no-OVA) for the different cytokines **(I)** (n= 4). Marker gate (highly positive cells) of the OVA sets for each cytokine was based on the corresponding no OVA control. An unstained cell histogram of each cytokine was used as a reference. Data presented as mean ± SEM. The exact p values are depicted in the figure using the Student’s unpaired t-test.

Having demonstrated the requirement of Wnt5A signaling in antigen processing and concomitant CD8 T cell response *in vitro*, we investigated if Wnt5A influences antigen-specific CD8 T cell activation *in vivo*, by examining the effect of Wnt5A on the antigen recall response by CD8 T cells in a mouse model. B6;129S7-*Wnt5a^tm1Amc^*/J mice (both Wnt5A+/+ and Wnt5A+/-), where Wnt5A heterozygosity-related diminution in Wnt5A expression has already been demonstrated (53), were used for this purpose. We compared the recall response to OVA antigen in both groups of mice after immunization with OVA. The FACS gating strategy for assessing CD8 T cell recall response is illustrated in **Figure S11A**. In essence, following OVA immunization with two doses of the antigen, both sets of mice, Wnt5A wild-type and Wnt5A heterozygous were sacrificed, and CD8 T cell recall response to antigen was evaluated by flow cytometry after stimulating splenocyte-lymph node cultures derived from each set with fresh OVA antigen following CFSE labeling. For each CFSE labeled OVA pulsed splenocyte-lymph node culture, a similar labeled “no OVA” culture was used as reference. CD8 T cell proliferation was measured after 4 days, through analysis of CFSE dye dilution in FACS dot plot on live CD3**^+^** CD8**^+^** gated cells after live/dead cell gating, as represented by specific examples (**Figure 5A**). We observed significantly lower CD8 T cell proliferation in response to OVA stimulation in the splenocyte-lymph node cultures of the Wnt5A heterozygous (HET) mice as compared to those of the corresponding wild-type (WT) mice. To further ensure the specificity of CD8 T cell response during recall response to OVA, we used labeled SIINFEKL bound MHCI tetramer as a tool for identification of OVA-specific CD8 T cells in a subset of the experiments represented by **Figure 5A**. We noted that the percentage of SIINFEKL-MHCI tetramer bound CD8 T cells from the proliferated (CFSE less) CD8 T cell population was relatively less in the Wnt5A heterozygous mice as compared to that observed for the wild-type controls (**Figure 5B**). Analysis of different sets of similarly stimulated splenocyte-lymph node cultures for CD8 T cell proliferation and specificity for the SIINFEKL-MHCI tetramer indicated that OVA-specific CD8 T cell recall response after immunization is compromised in the Wnt5A heterozygous background (**Figures 5C, D**). Furthermore, the percentage of CD44^high^CD62L^low^ phenotype among the proliferating MHC tetramer binding CD8 T cells was also significantly low in the Wnt5A heterozygous mice in comparison to the wild-type, as evaluated by the FACS quadrant plot (**Figures 5E, F**), indicating compromised differentiation to an effector memory phenotype during the recall response to antigen (54). In compliance with the compromised CD8 T cell response to OVA, splenic CD11c+ cells, of the Wnt5A heterozygous mice also exhibited lesser efficiency in the processing of OVA as compared to the wild-type mice (**Figure 5G**), indicating that at least a certain fraction of splenic CD11c+ cells in the heterozygous mice are defective in antigen processing. The corresponding gating strategy is explained in **Figure S11B**.

Along the same line of investigation, T cell activation was evaluated by comparing the levels of IFNγ, IL2 and granzyme B (50–52, 55). Increased OVA-specific CD8 T cell proliferation in the wild-type splenocyte-lymph node cultures as compared to the heterozygous sets correlated with higher percentages of OVA-specific CD8 T cells with augmented IFNγ, IL2, and GRB levels as demonstrated by FACS (**Figures 5H, I**) implying that a higher percentage of activated OVA-specific CD8 T cells is present in the wild-type sets. The FACS gating strategy for cytokines is depicted in **Figure S11C**. Overall, these results indicate that OVA antigen-specific CD8 T cell priming/sensitization is markedly better in the Wnt5A wild-type mice than their heterozygous counterparts. The lack of appropriate CD8 T cell priming in the Wnt5A heterozygous mice could very well be an outcome of ineffective antigen processing and presentation.

## Discussion

Processing of exogenous antigens by antigen-presenting cells and concomitant CD8 T cell activation are vital aspects of cell-mediated immunity and buildup of CD8 T cell memory. Thus impairments in antigen processing and CD8 T cell activation are linked to susceptibility to several infections (2, 3). Although there has been much research performed and extensive knowledge gained along these lines, several questions remain in relation to how the processing and presentation of exogenous antigens toward the generation of antigen-responsive CD8 T cells for future recall response to an antigen are coordinated.

In this study, we evaluated the role of Wnt5A signaling in antigen-processing/presentation and CD8 T cell activation in light of its documented involvement in cytoskeletal actin remodeling, a core element in the coordination of intracellular processes in the backdrop of cell activation by external stimuli (4–6, 11). In order to precisely focus on host factors that direct antigen processing/presentation toward T cell activation, we avoided the use of external stimulatory factors such as LPS in our experimental setup.

Initially, using mouse BMDC as antigen-presenting cells and OVA as a model antigen we demonstrated that Wnt5A signaling in antigen-presenting cells supports antigen processing and antigen-responsive CD8 T cell activation by regulating both actin and proteasome dynamics. Blockade of Wnt5A signaling through Wnt5A siRNA transfection caused disruption in the regulation of actin and proteasome assembly in the context of the incumbent antigen leading to impairment in antigen processing/presentation and CD8 T cell activation (**Figures 1**-**Figure 4**). In light of the association of proteasome activity with actin dynamics (56), Wnt5A-directed effects on actin and proteasome regulation in the context of antigen processing/presentation may very well be interconnected. Although not demonstrated in this study, the observed influence of Wnt5A on PA28 α/β expression could be mediated through IFNγ, which has been previously found by us to be regulated by Wnt5A signaling (29).

Impairment in the activation of antigen-responsive CD8 T cells was additionally observed in a Wnt5A depleted background in Wnt5A heterozygous (Wnt5A+/-) mice as illustrated by their significantly reduced CD8 T cell recall response to OVA antigen in comparison to the wild-type cohorts, following immunization with OVA. Inhibited cross-presentation of OVA to CD8 T cells in the heterozygous mice was evident from the reduced numbers of SIINFEKL-MHCI tetramer-bound activated CD8 T cells during the recall response to OVA. On the whole, these results suggest that host Wnt5A signaling supports antigen priming of CD8 T cells through antigen processing and cross-presentation, and as a corollary, facilitates the generation of antigen-specific memory that is reflected in the recall response to antigen (**Figure 5**). In line with these findings, and the already documented role of Wnt5A signaling in resisting Chandipura virus infection in macrophages (29), it will be important to investigate if the inadequate cell-mediated immune response and antigen-specific memory in the Wnt5A heterozygous mice render these susceptible to viral infections. Wnt5A heterozygous mice are susceptible to polymicrobial sepsis (5), but it is not clearly known if this is due to defects in CD8 T cell-mediated immunity.

The outcome of Wnt signaling on T cell response is to a large extent dependent on the pathophysiological context. Wnt5A has been reported to suppress the adaptive immune response of CD8 T cells in melanoma through metabolic programming (57). Wnt1 and Wnt3A have also been shown to have regulatory effects on immunity in different tumor microenvironments (58, 59). In this study, using both BMDC and a mouse model we indicated that steady state Wnt5A signaling supports the processing of exogenous antigen in coordination with actin and proteasome dynamics to guide cross-presentation and antigen-specific CD8 T cell activation, which should be useful for future recall response to antigen.

To our knowledge, this study is the first to describe the role of Wnt5A-mediated coordination of actin and proteasome dynamics in antigen processing and presentation under steady-state conditions. Future studies in higher organisms directed toward the identification of host factors such as Wnt5A that have significant involvement in the processing of exogenous antigens and enrichment of antigen-responsive CD8 T cells should prove useful in unveiling the unsolved intricacies of cell-mediated immunity. Such studies should also aid in the design of effective vaccines. There are, however, certain limitations in our study that need to be recognized. Although a requirement of Wnt5A signaling in antigen-specific CD8 T cell activation has been demonstrated, the efficiency of the CD8 T cells in antigen-specific lysis of target remains unanalyzed, limiting functional assessment. There is a dearth of information on the role of CD4 T cells in the Wnt5A-dependent cell-mediated response to antigenic stimulus. Moreover, the influence of Wnt5A signaling on B cell activation and antibody response to antigen, another vital feature of immunity remains to be evaluated.

## Data availability statement

The original contributions presented in the study are included in the article/**Supplementary Material**. Further inquiries can be directed to the corresponding author.

## Ethics statement

The animal study was reviewed and approved by Animal Ethics Committee of CSIR-IICB (CSIR-IICB-AEC).

## Author contributions

MS designed research, analyzed data, and wrote the paper. TRS performed research, contributed to research design, analyzed data, and assisted in writing the paper. All authors contributed to the article and approved the submitted version.

## Funding

This work was supported by (BT/PR7106/MED/29/639/2012) from the Department of Biotechnology, Govt. of India, and institutional funding P-07. TRS was supported by the CSIR fellowship.

## Acknowledgments

The authors thank Tanmoy Dalui for FACS; Shounak Bhattacharya for confocal microscopy; CSIR-IICB central instrument facility for instrument support; CSIR-IICB animal house facility for animal breeding & maintenance; Shreyasi Maity for helping in animal handling; Soham Sengupta, Ananya Ganguly, Deepesh Kumar Padhan for general lab support; and Indrajit Sikder for technical assistance.

## Conflict of interest

The authors declare that the research was conducted in the absence of any commercial or financial relationships that could be construed as a potential conflict of interest.

## Publisher’s note

All claims expressed in this article are solely those of the authors and do not necessarily represent those of their affiliated organizations, or those of the publisher, the editors and the reviewers. Any product that may be evaluated in this article, or claim that may be made by its manufacturer, is not guaranteed or endorsed by the publisher.
