# Supplementary material for: Wnt5A signaling supports antigen processing and CD8 T cell activation
Source: Front Immunol. 2022 Aug 26;13:960060. doi: 10.3389/fimmu.2022.960060 (PMC9459031; doi:10.3389/fimmu.2022.960060)
Supplement: Supplementary file 1 [file DataSheet_1.pdf]

Supplementary figure 1

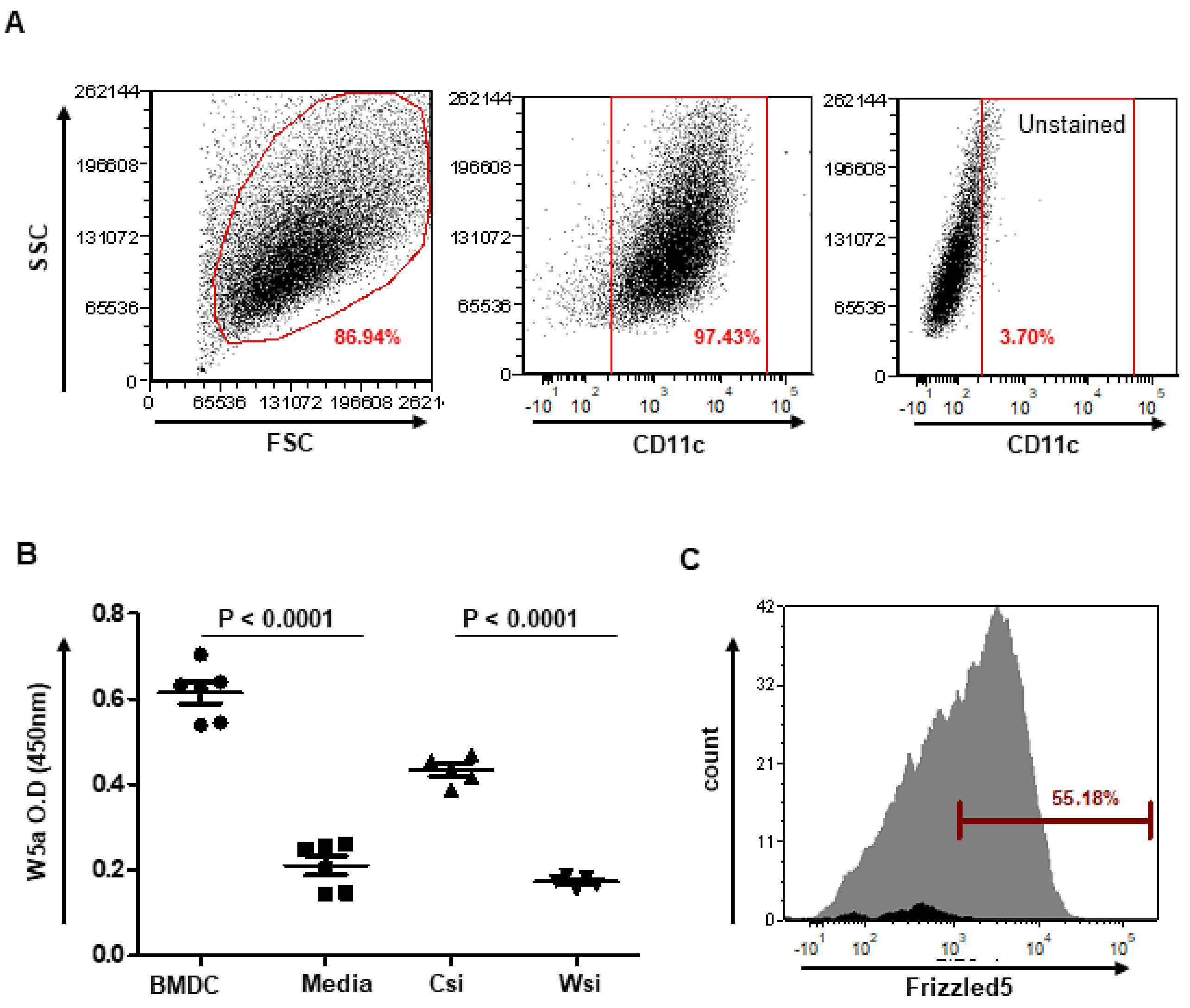

Figure S1: <u>Bone marrow-derived dendritic cells (BMDC) express Wnt5A and Frizzled5 (Wnt5A receptor)</u>: A: Bone marrow-derived dendritic cells were generated from BALB/c mice bone marrow and stained with CD11c antibody to determine purity. FACS dot plot shows the percentage of CD11c positive BMDC. Gating was based on unstained cell dot plot. **B:** ELISA with anti-Wnt5A antibody demonstrating secretion of Wnt5A (W5a) from BMDC as corroborated by its reduction through Wnt5A siRNA (Wsi) mediated depletion of Wnt5A. Cell culture media without BMDC and scramble siRNA (control Csi) transfected cells are used as references for untransfected BMDC and Wnt5A siRNA transfected BMDC respectively. **C:** FACS histogram demonstrating Frizzled5 surface expression on BMDC; secondary antibody control (black histogram) is the reference.

Supplementary figure 2

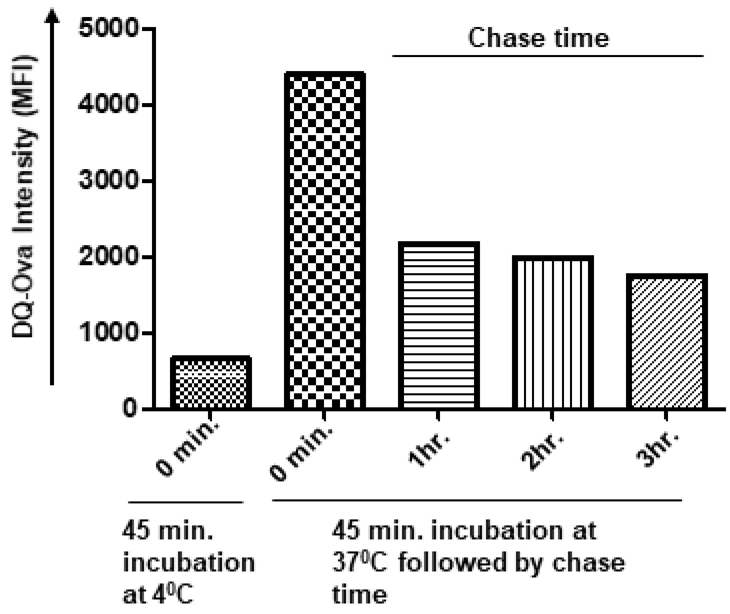

Figure S2: FACS based MFI plot demonstrating DQ-OVA processing in BMDC. BMDC was incubated for 45 min. with 5ug/ml DQ-Ova at 37⁰C and after chase time (0 min. – 3hr.), MFI was measured by FACS. Maximum DQ-OVA fluorescence (as a measure of proteolytic cleavage) was obtained after 45 min. at 37⁰C. 4⁰C incubation was used as a negative control. MFI: Mean Fluorescence Intensity (Geometric Mean).

Supplementary figure 3

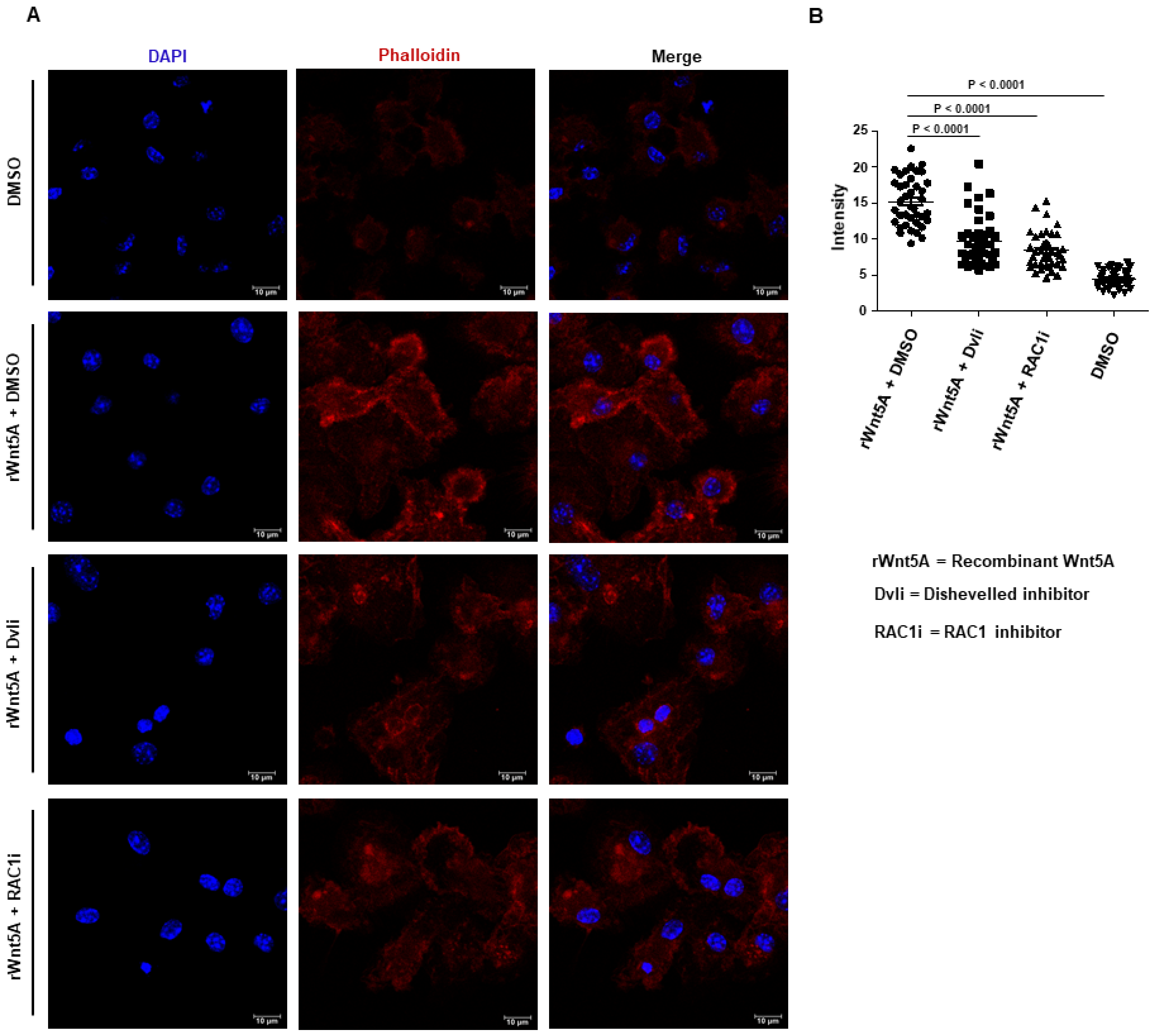

Figure S3: **<u>Inhibition of Wnt5A induced actin assembly in BMDC by Wnt5A signaling inhibitors.</u>** **A:** Confocal microscopy demonstrating phalloidin (Red) staining as a marker of actin assembly in Wnt5A conditioned BMDC in the presence and absence of Disheveled and RAC1 inhibitors (n= 2). **B:** Graphical representation of phalloidin intensity as estimated by ImageJ, each dot in the plot representing a cell. DAPI (blue) represents nuclei.

Supplementary figure 4

A

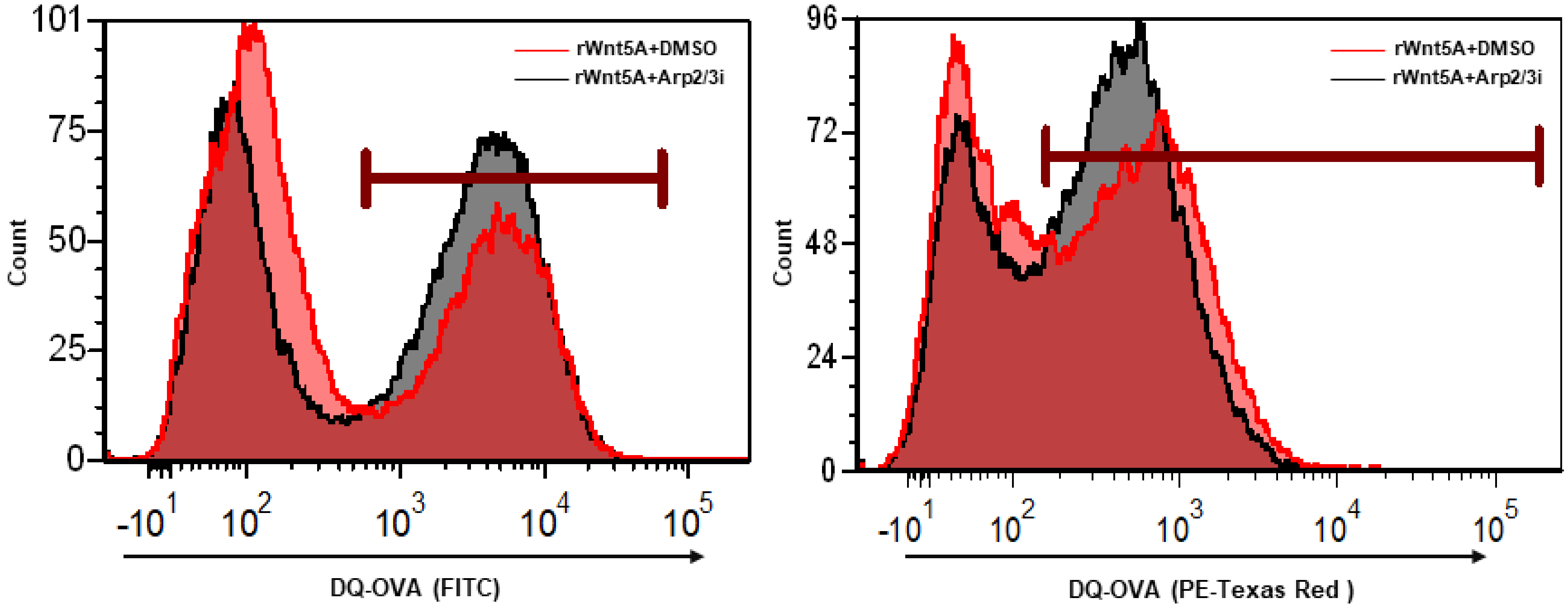

B

Arp2/3i = Arp2/3 inhibitor

rWnt5A = Recombinant Wnt5A

Geometric mean			
DQ-OVA (FITC)		DQ-OVA (PE-Texas Red)	
rWnt5A+DMSO	rWnt5A+Arp2/3i	rWnt5A+DMSO	rWnt5A+Arp2/3i
4222.89	3787.89	628.30	519.30

Figure S4: **<u>Inhibition of DQ-OVA processing by Arp2/3 inhibitor.</u>** **A:** FACS histogram represents DQ-OVA processing in BMDC in the FITC channel and PE-Texas Red channel in the presence (black) and absence (red) of Arp2/3 inhibitor, which blocks actin assembly (n= 2). **B**: The table depicts geometric mean values for DQ-OVA processing in different conditions.

Supplementary figure 5

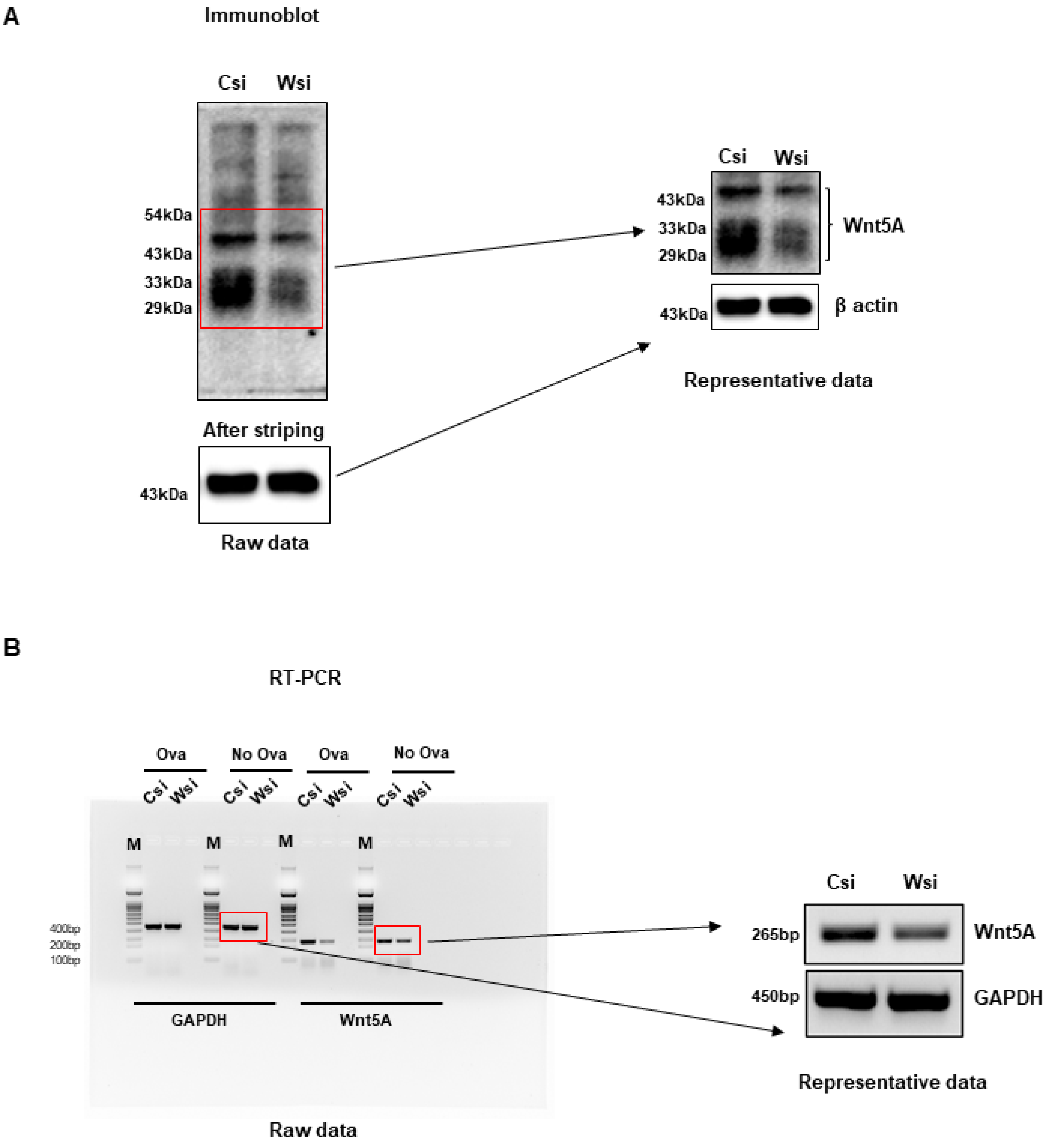

Figure S5: <u>Representation of raw data related to Figure 1</u>: A: Representation of Wnt5A immunoblot (cropped) from corresponding raw data. Multiple bands indicate the presence of different transcript variants and/or post-translational modifications of Wnt5A. **B:** Representation of RT-PCR (cropped) from corresponding raw data. M: DNA Ladder

Supplementary figure 6

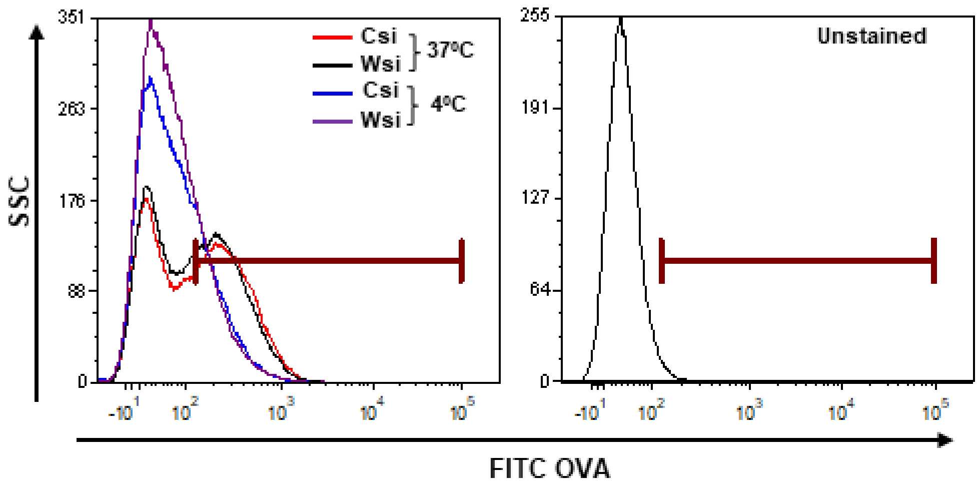

Figure S6: <u>Wnt5A does not influence OVA uptake by BMDC significantly</u>. FITC OVA uptake was not significantly influenced by Wnt5A depletion as shown by FACS histogram. There was no significant difference in FITC OVA (5ug/ml) uptake by Wnt5A siRNA (Wsi) and control siRNA (Csi) transfected BMDC following FITC OVA incubation for 45 min. at either 37⁰C or 4⁰C. Gating was based on unstained cell histogram.

Supplementary figure 7

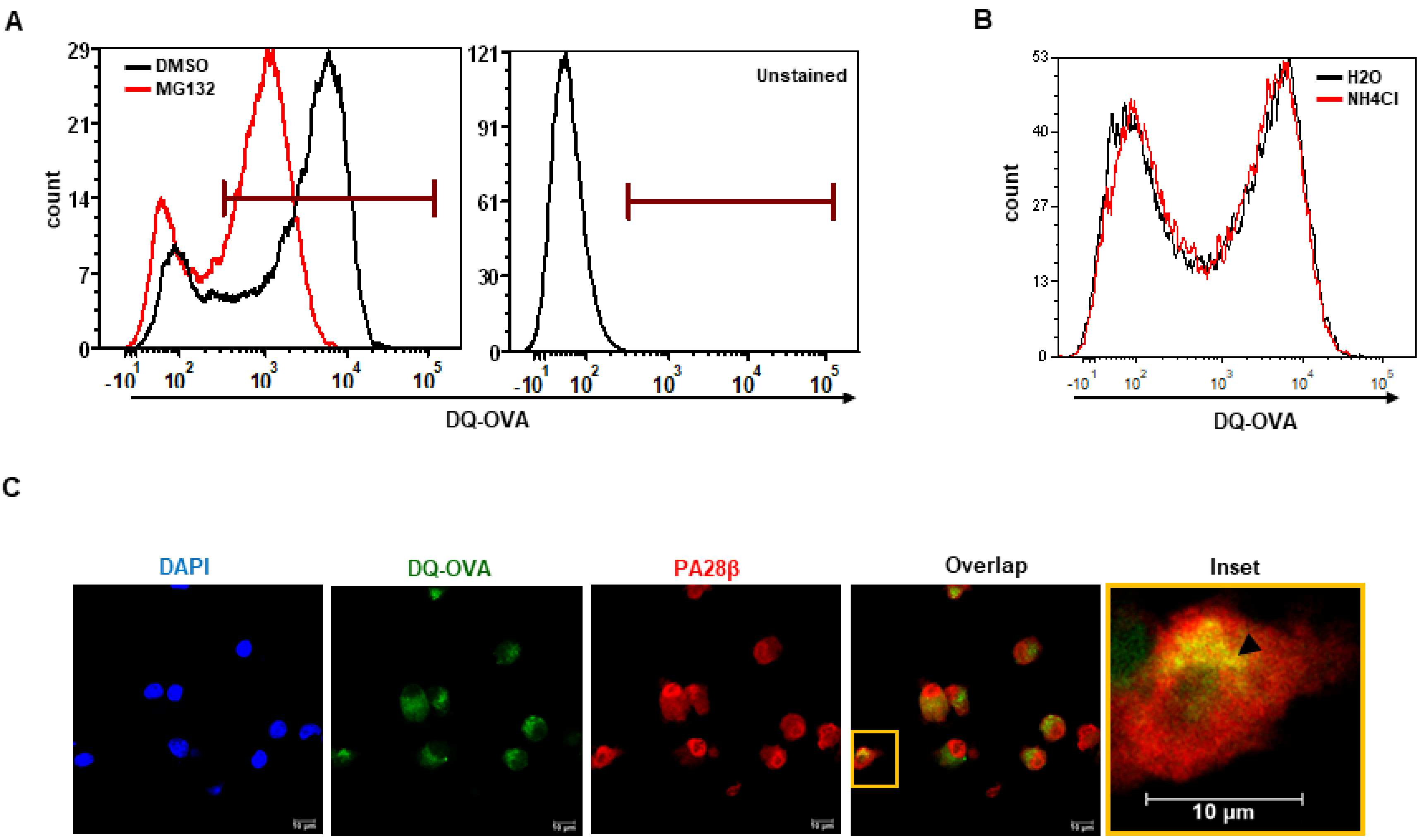

Figure S7: <u>DQ-OVA processing in BMDC is proteasome-mediated:</u> A: FACS demonstrating reduction in DQ-OVA fluorescence in BMDC after MG132 (proteasome inhibitor) treatment (10uM) for 30 min. as compared to control (DMSO: vehicle control treatment). Marker gate was based on unstained cell histogram. **B:** FACS histogram showing unchanged DQ-OVA fluorescence in BMDC in presence of NH4Cl. **C:** DQ-OVA co-localizes with PA28β (proteasome regulator) in BMDC as demonstrated by confocal microscopy. DAPI (blue) denotes nuclei. Arrow mark (black) shows overlap (yellow spots).

Supplementary figure 8

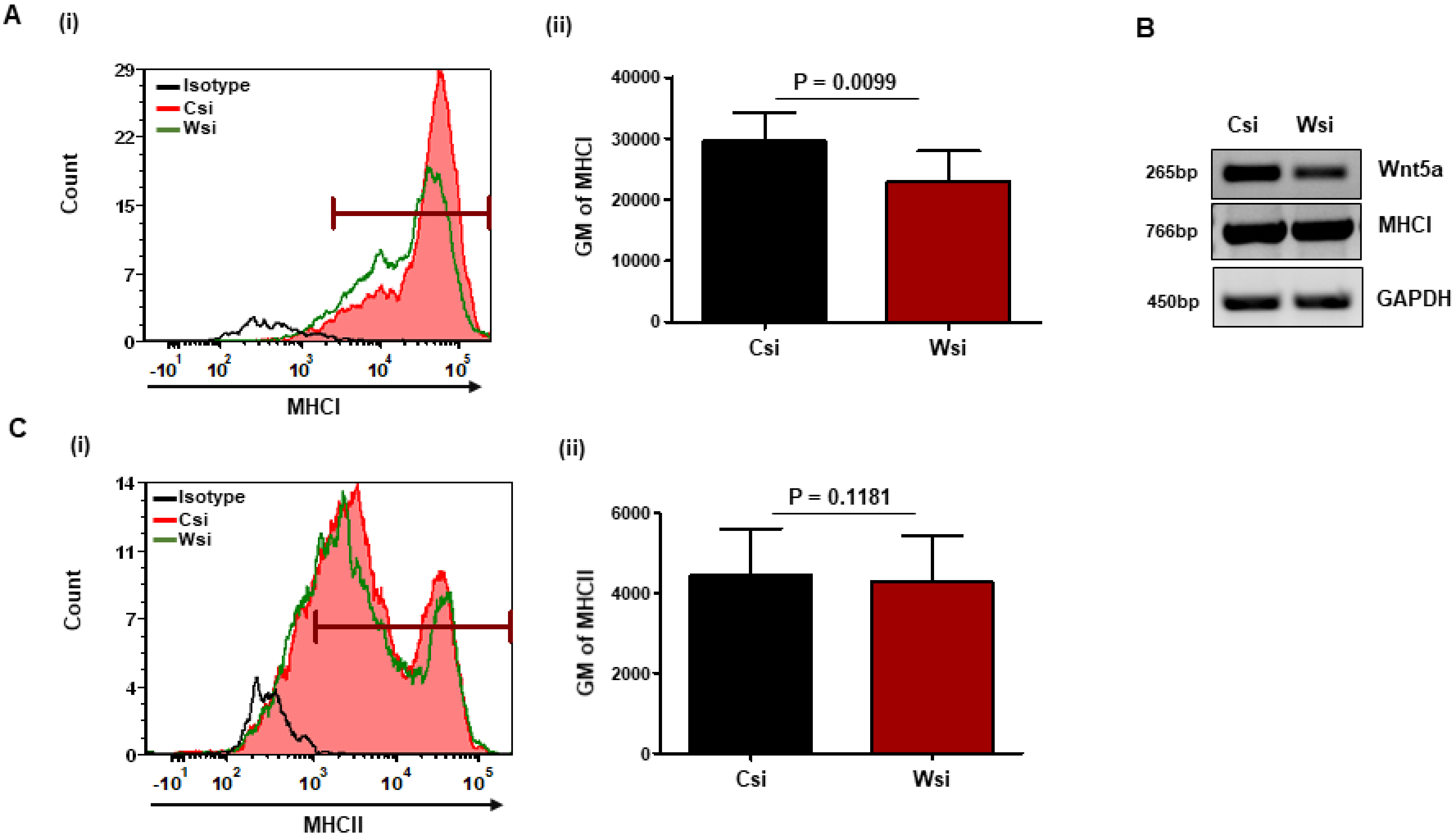

Figure S8. <u>Surface expression of MHC class I in BMDC is Wnt5A dependent.</u> A: FACS histogram (i) and MFI (GM) graph (ii) showing decrease in surface expression of MHCI in Wnt5A siRNA transfected BMDC (Wsi) as compared to control (Csi) after OVA pulsing. BMDC is gated on CD11c. **B**. RT-PCR analysis shows that MHCI gene expression in BMDC is not changed by Wnt5A depletion through siRNA transfection. **C.** MHCII surface expression is not significantly affected by Wnt5A depletion. Black histogram represents the isotype control.

Supplementary figure 9

A

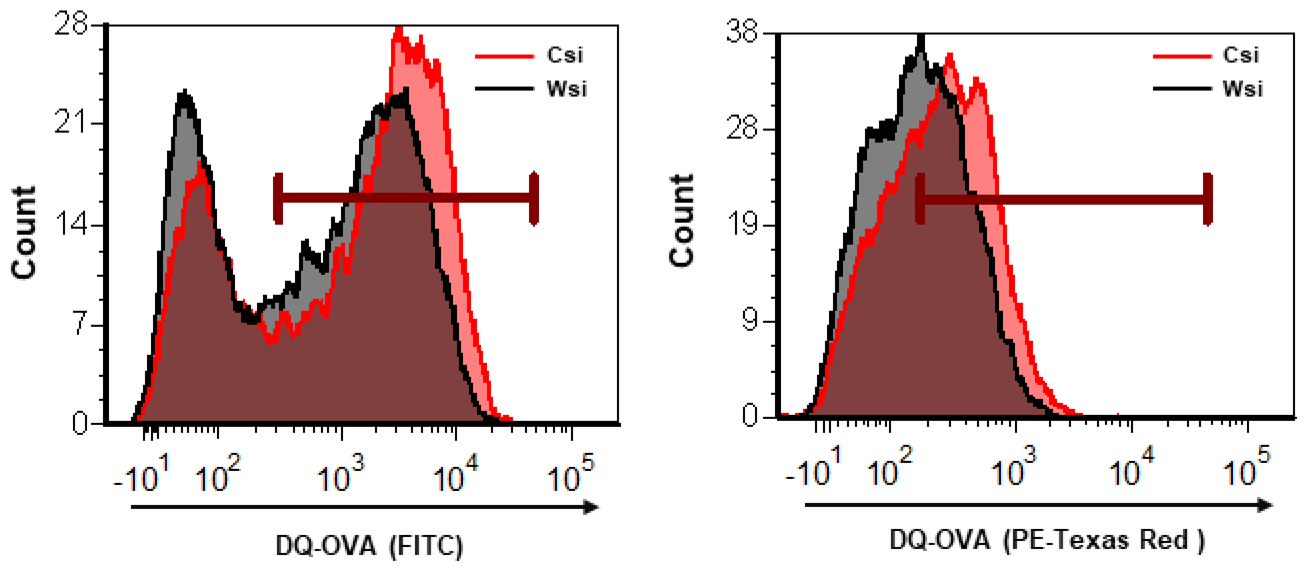

B

Geometric mean			
DQ-OVA (FITC)		DQ-OVA (PE-Texas Red)	
Csi	Wsi	Csi	Wsi
2672.49	1962.93	403.19	335.05

Csi = Control siRNA

Wsi = Wnt5A siRNA

C D E F

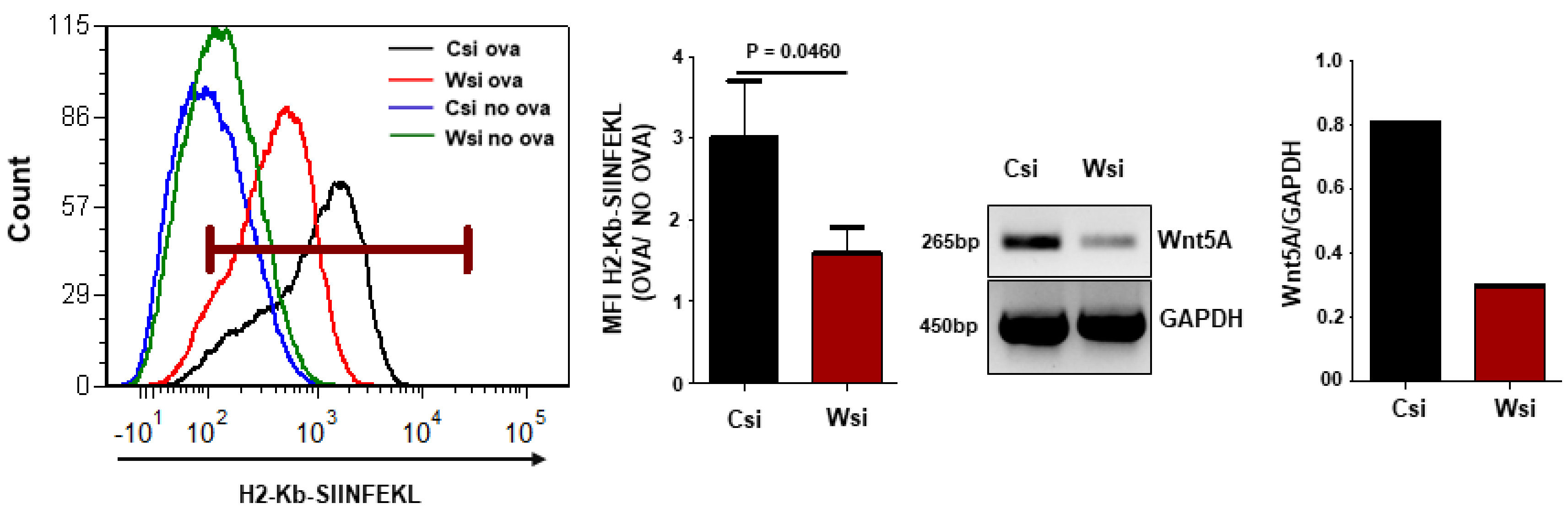

Figure S9: <u>Reduction of Wnt5A expression in BMDC by siRNA transfection reduces OVA processing and presentation.</u> A: FACS histogram represents reduced DQ-OVA processing in Wnt5A siRNA (Wsi) transfected BMDC as compared to control (Csi) in the FITC and PE-Texas Red channels. **B:** Table depicts geometric mean values of DQ-OVA fluorescence. **C, D:** FACS histogram (C) and corresponding graphical representation (D) of increased OVA-specific H2-Kb-SIINFEKL surface expression (MFI: GM) in control siRNA (Csi) with respect to Wnt5A siRNA (Wsi) sets (n= 4). **E, F:** RT-PCR (E) and corresponding densitometry graph (F) showing the decrease in Wnt5A expression upon Wnt5A siRNA transfection.

Supplementary figure 10

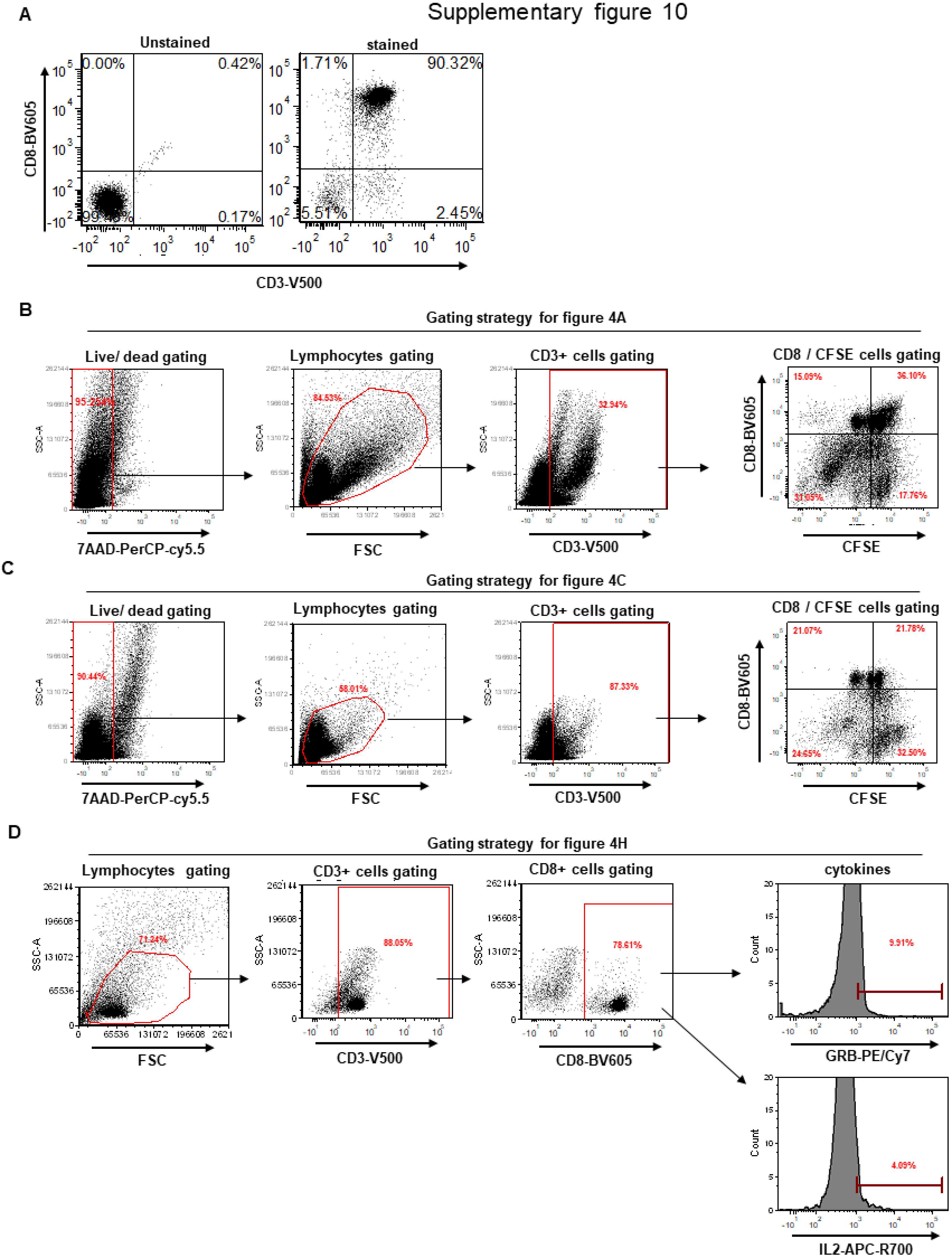

Figure S10: **<u>OVA-sensitized CD8 T cell proliferation and activation upon exposure to antigen-pulsed BMDC</u>**. **A**: Representation of CD8 T cell purity. **B**, **C**: Representation of gating strategy for CFSE^{dim} CD3+CD8+ proliferating T cells after coculture with OVA pulsed BMDC. **D**: Representation of gating strategy for IL2^{high} GRB^{high} CD3+CD8+ T cells. IL2^{high} and GRB^{high} cells are gated based on "no OVA" condition as reference.

Supplementary figure 11

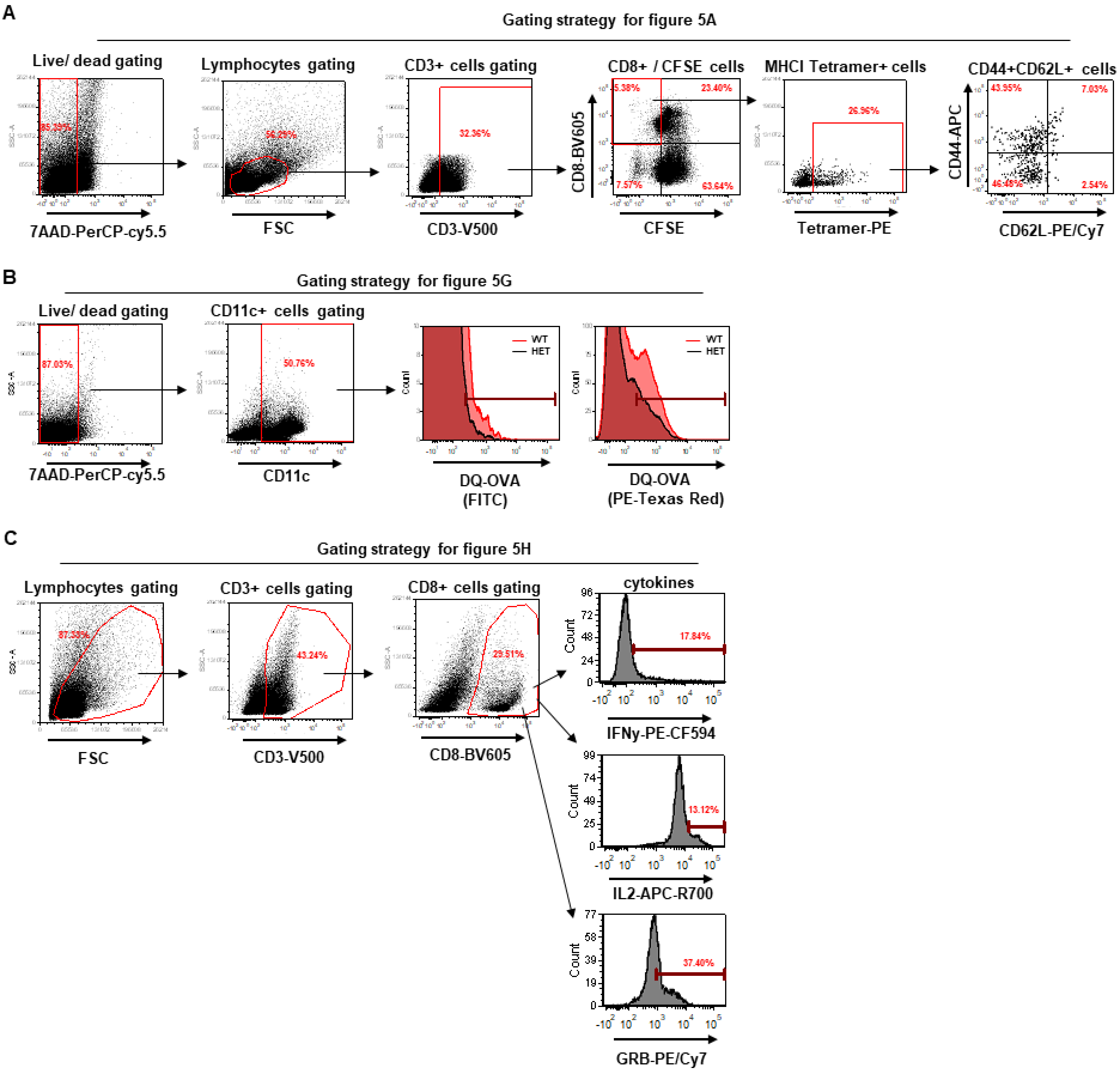

Figure S11: <u>OVA antigen recall response by CD8 T cells after OVA immunization correlates with OVA processing</u>. A: Representation of gating of live CD3+CD8+ OVA SIINFEKL responsive T cells after restimulation of harvested splenocytes with OVA. **B**: Representation of gating of live CD11c+ cells exhibiting DQ-OVA fluorescence as a measure of OVA processing. **C**: Representation of gating of IFNγ^{high}, IL2^{high} and GRB^{high} CD3+CD8+ T cells. Gating for high cytokine producers is based on “no OVA” condition as reference.

Supplementary Table 1

Reagent	Company	Catalogue No.
7-AAD cell viability stain	Biolegend	420403
Albumin from chicken egg	Sigma-Aldrich	A5503-1G
Alexa fluor 555 phalloidin	Invitrogen	A34055
Anti-human/mouse Wnt5A monoclonal antibody	R & D Systems	MAB645
anti-PA28α	Santa Cruz Biotechnology	sc-21267
Anti-PA28ß	Biobharati Life Sciences	BB-AB0190
anti-rat IgG-HRP	R & D Systems	HAF005
APC rat anti-mouse CD44	BD Biosciences	559250
ANTI-MO SIINFEKL 25D1.16 PE,	Invitrogen	12-5743-81
APC-hamster anti-mouse CD11c	BD Biosciences	550261
APC-R700 rat anti-mouse IL-2	BD Biosciences	565186
Arp2/3 complex Inhibitor I	Merck	CK-666
Bradford reagent	Biorad	5000006
Brefeldin	Calbiochem	203729-1MG
BV605-rat anti-mouse CD8	BD Biosciences	563152
cDNA synthesis kit	Biobharati Life Sciences	BB-E0045
CFSE	Invitrogen	C34570
Complete Freund‘s Adjuvant (CFA)	Difco	263810
control siRNA	Eurogentec/ Dharmacon	SR-CL000-005/ D-001206-13-05
DAPI	Invitrogen	D1306
donkey anti-goat alexa fluor 488	eBioscience	A-11055
donkey anti-goat alexa fluor 546	eBioscience	A-11056
donkey anti-rabbit alexa fluor 546	eBioscience	A10040
Dishevelled inhibitor	Merck	CAS294891-81-9
DQ-OVA	Invitrogen	D12053
Fetal Bovine Serum	Invitrogen	10082147

FITC-OVA	Invitrogen	023020
GM-CSF	Peprotech	315-03
H-2Kb OVA Tetramer-SIINFEKL-PE	Medical & Biological Laboratories	TS-5001-1C
IL4	Peprotech	214-14
Incomplete Freund‘s Adjuvant (IFA)	Difco	263910
L-Glutamine	Invitrogen	25030081
Lipofectamine RNAimax	Invitrogen	56532
MG132	Millipore	474790
Mini Separator (Macs) Columns	Miltenyi Biotec	130-042-201
Mouse CD8a+ isolation kit	Miltenyi Biotec	130-104-075
OptiMEM	Invitrogen	31985-070
OVA SIINFEKL peptide (257-264)	Anaspec	AS-60193-1
PCR kit	Biobharati Life Sciences	BB-E0010S
PE-Cyanine 7-rat anti-mouse Granzyme B	eBioscience	25-8898-82
PE-IgG2a k isotype	BD Biosciences	553457
PE-mouse anti-mouse H-2K[d]	BD Biosciences	553566
PenStrep	Invitrogen	15140122
Per CP-Cy 5.5 rat IgG2b k isotype	BD Biosciences	550764
Per CP-Cy 5.5-rat anti-mouse I-A/I-E	BD Biosciences	562363
Poly-l-lysine	Sigma-Aldrich	P-8920
Prolong glass antifade	Invitrogen	P36982
Propidium iodide (PI)	BD Biosciences	51-66211E
Purified chicken ovalbumin (OVA, grade VI)	Sigma-Aldrich	A5503-1G
rat anti-mouse CD62L	BD Biosciences	553148
Recombinant Wnt5A	Millipore	GF146
RPMI 1640	Invitrogen	31800-022
RAC1 inhibitor	Merck	NSC23766

TMB solution	Merck	CL07-1000MLCN
TRIzol reagent	Invitrogen	15596018
Tween-20 detergent	Merck	655205-250ML
V500-Syrian hamster anti-mouse CD3	BD Biosciences	560771
Western HRP substrate	Millipore	WBLUC0500
Wnt5A siRNA	Eurogentec/ Dharmacon	SR-NP001-001/ M-065584-00-0005

Table 1: List of reagents used in the study.
